# Tumor cell-derived SPON2 promotes M2-polarized tumor-associated macrophage infiltration and cancer progression by activating PYK2 in CRC

**DOI:** 10.1186/s13046-021-02108-0

**Published:** 2021-09-28

**Authors:** Chengmei Huang, Ruizhang Ou, Xiaoning Chen, Yaxin Zhang, Jiexi Li, Yihao Liang, Xiaohui Zhu, Lei Liu, Mingzhou Li, Dagui Lin, Junfeng Qiu, Guanglong Liu, Lingjie Zhang, Yuanyuan Wu, Huiyi Tang, Yanmin Liu, Li Liang, Yanqing Ding, Wenting Liao

**Affiliations:** 1 Department of Pathology, Nanfang Hospital, Southern Medical University, Guangzhou, 510515 Guangdong China; 2 Department of Pathology, School of Basic Medical Sciences, Southern Medical University, Guangzhou, 510515 China; 3 Guangdong Provincial Key Laboratory of Molecular Tumor Pathology, Guangzhou, Guangdong China; 4 Department of Cancer Biology, The University of Texas MD Anderson Cancer Center, Houston, TX 77030 USA; 5 Department of Orthopedist, Guangdong Provincial Hospital of Chinese Medicine, Guangzhou, 510000 China; 6 State Key Laboratory of Oncology in South China, Collaborative Innovation Center for Cancer Medicine, Sun Yat-Sen University Cancer Center, Guangzhou, China; 7 Department of Histology and Embryology, School of Basic Medical Sciences, Southern Medical University, Guangzhou, 510515 China

**Keywords:** SPON2, Colorectal cancer, Tumor-associated macrophages, Invasion, Metastasis, PYK2

## Abstract

**Background:**

Tumor-associated macrophages (TAMs) are key regulators of the complex interplay between cancer and the immune microenvironment. Tumor cell-derived spondin 2 (SPON2) is an extracellular matrix glycoprotein that has complicated roles in recruitment of macrophages and neutrophils during inflammation. Overexpression of SPON2 has been shown to promote tumor cell migration in colorectal cancer (CRC). However, the mechanism by which SPON2 regulates the accumulation of TAMs in the tumor microenvironment (TME) of CRC is unknown.

**Methods:**

Immunohistochemistry was used to examine SPON2 expression in clinical CRC tissues. In vitro migration assays, transendothelial migration assays (iTEM), and cell adhesion assays were used to investigate the effects of SPON2 on monocyte/macrophage migration. Subcutaneous tumor formation and orthotopic implantation assays were performed in C57 BL/6 mice to confirm the effects of SPON2 on TAM infiltration in tumors.

**Results:**

SPON2 expression is positively correlated with M2-TAM infiltration in clinical CRC tumors and poor prognosis of CRC patients. In addition, SPON2 promotes cytoskeletal remodeling and transendothelial migration of monocytes by activating integrin β1/PYK2 axis. SPON2 may indirectly induce M2-polarization through upregulating cytokines including IL10, CCL2 and CSF1 expression in tumor cells. Blocking M2 polarization and Macrophage depletion inhibited the SPON2-induced tumors growth and invasion. Furthermore, blocking the SPON2/integrin β1/PYK2 axis impairs the transendothelial migration of monocytes and cancer-promoting functions of TAMs in vivo.

**Conclusions:**

Our findings demonstrate that SPON2-driven M2-TAM infiltration plays an important role during CRC tumor growth and metastasis. SPON2 may be a valuable biomarker guiding the use of macrophage-targeting strategies and a potential therapeutic target in advanced CRC.

**Supplementary Information:**

The online version contains supplementary material available at 10.1186/s13046-021-02108-0.

## Background

Colorectal cancer (CRC) is the third most common cancer in the world [1]. Metastasis is the major cause of cancer morbidity and mortality in CRC. Although novel therapeutic choices have been improved, the five-year survival of patients with metastatic CRC remains only approximately 14% [2]. The tumor microenvironment (TME) has been shown to play an essential role in CRC progression and metastasis [3]. Understanding the components of the TME and their interplay with tumor cells is helpful for developing new strategies against metastatic CRC.

Tumor-associated macrophages (TAMs) are prominent tumor-infiltrating immune cells in the TME that suppress antitumor immunity and foster tumor progression [4–6]. Infiltration of TAMs is associated with a poor prognosis in cancer patients [7, 8]. However, the role of TAMs in CRC is controversial. Studies have reported that TAMs are beneficial to the prognosis of patients [9, 10], and most of these studies analyzed TAMs without considering the heterogeneity of these macrophages (e.g., distinct pro- or anti-inflammatory subpopulations (M1-TAMs and M2-TAMs) [11] and their spatial distribution within tumors [12]. Many recent studies have provided strong evidence that TAMs facilitate CRC growth and progression [13–17]. Most macrophages have the tendency to polarize into an M2-like state in tumors with advanced stages [18, 19]. In addition, most of the macrophages located at the invasive front of advanced CRC tumors display the M2-TAM phenotype [12]. However, how tumor cells affect TAM accumulation and their pro-tumoral phenotype in invasive CRC has not yet been well established.

TAMs are classically thought to be derived from peripheral blood monocytes [20–22]. Monocytes are recruited to tumors by chemokines (e.g., CCL2), cytokines (e.g., colony-stimulating factor-1 (CSF-1)), and their complement cascade [23, 24]. They extravasate from the peripheral circulation and differentiate into TAMs in the TME and are polarized into M2 macrophages by cytokines (e.g., CCL2, CSF1, IL10 and CCL5) [25–27]. IL10 has been detected in the tumor microenvironment of many cancer types and has been thought to promote tumor immune escape by polarizing TAMs to the M2 phenotype and inhibiting the functions of antigen presenting cells [28–30]. However, it has been reported to have anti-tumor effects with immune-dependent mechanisms, including activation of CD8 + T cells (CTLs) [31]. Therefore, it will be necessary to evaluate a potential therapeutic intervention by either inhibiting or promoting the IL10 pathway on a case-by-case basis in specific cancer types and patient subpopulations. Inhibition of CCL2/CCR2 signaling blocks the recruitment of inflammatory monocytes and reduces metastasis in mouse models of breast cancer, hepatocellular cancer, and prostate cancer [24, 32–34]. However, suppression of CCL2 expression only leads to a transient reduction in myeloid cell recruitment and a temporary delay in metastatic tumor growth in a mouse model of CRC [35]. Similarly, CSF-1 inhibitory antibodies or pharmacological inhibition of the CSF-1/CSF-1R axis effectively blocked the recruitment of macrophages at tumor sites [36]. However, the degree of CSF1R signaling dependency of macrophages at different locations is unclear and the clinical translation of depleting TAMs by targeting CSF-1/CSF1R is limited [37]. Therefore, the regulation of TAM accumulation and their function in invasive CRC must be comprehensively understood and the efficiency of current therapies must be improved.

SPON2 (spondin-2, Mindin, DIL-1) is a member of the F-spondin family of secreted ECM proteins [38]. It is a host innate immune regulator and represents a unique pattern-recognition molecule in the ECM for microbial pathogens [39]. In hepatocellular carcinoma, SPON2 promotes M1-like macrophage recruitment and inhibits tumor metastasis [40]. In contrast, SPON2 is overexpressed in the serum or tissue samples of malignant tumors, such as ovarian cancer [41] and prostate cancer [42]. Functionally, overexpression of SPON2 in CRC cells increases cell motility and CRC metastasis in mice [43]. The distinct effect of SPON2 on metastasis in hepatocellular carcinoma and in CRC is probably due to the discrepancy in macrophage infiltration in the two types of tumors. In the current study, we demonstrated that tumor cell-derived SPON2 promotes the infiltration of TAMs with an M2-like phenotype and tumor metastasis in CRC. Mechanistically, SPON2 induces transendothelial migration of monocytes by activating PYK2.

## Materials and methods

### Cell lines

The human cell lines SW620, SW480, HCT116, DLD1, RKO, HUVEC and THP-1 and the mouse cell lines CT26, CMT93, MC38, RAW264.7 and C166 were preserved in the Department of Pathology, Southern Medical University, China. SW620 and SW480 cells were cultured in Leiboviz’s L-15 medium (Gibco) supplemented with 10% fetal bovine serum (FBS) (Gibco). HCT116 cells were cultured in McCoy’s 5A medium (Gibco) with 10% FBS. RKO and HUVEC cells were cultured in F-12 K medium (Gibco) with 10% FBS. DLD1, THP-1, CT26 and MC38 cells were cultured in RPMI 1640 medium (Gibco) with 10% FBS (Gibco). CMT93, RAW264.7 and C166 were cultured in Dulbecco's Modified Eagle's Medium (DMEM) (Gibco) supplemented with 10% FBS.

### Plasmids and generation of stably transfected cell lines

The SPON2 plasmid was generated by cloning PCR-amplified full-length human SPON2 cDNA into pCDH. For deletion of SPON2, 4 short hairpin RNA (shRNA) sequences were separately cloned into a pLKO.1 vector. The vectors pCDH and pLKO.1 were purchased from Addgene Inc. Transfection of plasmids was performed using Lipofectamine 2000 reagent (Invitrogen, Carlsbad, California, USA) according to the manufacturer’s instructions. Cells (2 × 10^5^) were seeded and infected by lentivirus generated by pCDH-SPON2-puro and pLKO.1-SPON2-shRNA-puro for 3 days. Stable cell lines expressing SPON2 and SPON2-shRNAs were selected with 1 µg/mL puromycin for 5 days. The primer sequences are provided in Supplementary Table S1.

### RNA isolation, reverse transcription (RT) and real-time PCR

Total RNA samples were extracted from the cultured cells using TRIzol reagent (Invitrogen) according to the manufacturer's instructions. Gene expression was analyzed by Q-RT-PCR using an ABI PRISM 7500 sequence detection system (Applied Biosystems, USA). For cDNA synthesis, 1 µg RNA was reverse transcribed using a reverse transcriptase kit (Vazyme). PCR amplification was performed using SYBR Green (Vazyme) in a total volume of 25 µl. The primer sequences used for amplification are provided in Supplementary Table S1.

### Western blotting analysis

Western blotting analysis was performed as previously described [44] using anti-SPON2 (A12077, ABclonal), anti-PYK2 (bs-3357R, Bioss), anti-p-PYK2 (bs-3400R, Bioss), anti-FAK (bs-1340R, Bioss), anti-Zyxin (60,254–1-Ig, Proteintech), anti-RhoA (HPA062346, Sigma-Aldrich), anti-cortactin (11,381–1-AP, Proteintech), anti-IL10 (20,850–1-AP, Proteintech), anti-CCL2 (66,272–1-Ig, Proteintech), and anti-CSF1 (14,779–1-AP, Proteintech) antibodies. Mouse monoclonal anti-α-tubulin antibody (RM2007, RayAntibody) was used as the internal control.

### Double immunohistochemistry staining and immunohistochemistry

Double immunohistochemistry staining was performed as previously described using SPON2 (Bioss, bs-11064R) and CD68 antibodies (ab213363, Abcam) [45]. Immunohistochemistry (IHC) staining was performed as previously described using SPON2 (bs-11064R, Bioss) and CD163 (ZM-0428, ZSGB-BIO) [46]. The degree of CD68 and CD163 IHC staining was reviewed and scored based on the proportion of positively stained stromal cells. The degree of SPON2 IHC staining was reviewed and scored independently by two observers based on both the proportion of positively stained tumor cells and the intensity of staining. The proportion of tumor cells was scored as follows: 0 (no positive tumor cells), 1 (< 10% positive tumor cells), 2 (10–50% positive tumor cells), and 3 (> 50% positive tumor cells). The intensity of staining was graded according to the following criteria: 0 (no staining); 1 (weak staining = light yellow), 2 (moderate staining = yellow brown), and 3 (strong staining = brown). The staining index was calculated as the staining intensity score × the proportion of positive tumor cells. Using this method of assessment, we evaluated the expression of SPON2 in benign colon epithelium and malignant lesions by determining the staining index based on scores of 0, 1, 2, 3, 4, 6, and 9. Cutoff values for SPON2 were selected on the basis of a measure of heterogeneity with the log-rank statistical test with respect to overall survival. Optimal cutoff values were identified: a staining index ≥ 4 was used to define tumors with high SPON2 expression, and an index ≤ 3 was used to define tumors with low SPON2 expression.

### Bone Marrow-Derived Macrophages (BMDM)

Bone marrow was isolated from femurs and tibias from C57 BL/6 mice using a 23- gauge needle. Cells were then cultured in RPMI 1640 supplemented with 10% FBS, 2.0mML-glutamine, 100 U/ml penicillin, 100 mg/ml streptomycin, non-essential amino acids, 14.2 mM b-mercaptoethanol, to which was added murine recombinant CSF1 (10 ng/ml; PeproTech, 351–02). Differentiating macrophages were cultured by transferring non-adherent cells 24 h after bone-marrow cells isolation and replenishing the medium with a fresh one every 48 h. BMDM differentiation was confirmed by flow cytometric evaluation of F4/80 expression. M0 macrophages (M0/M) were obtained by treating cultured cells with 10 ng/mL CSF1 for 5 days. Macrophages were then polarized to alternatively activated (M2/M) using 10 ng/mL IL4 (Peprotech, 214–14).

### Collection of conditioned media

To collect condition media (supernatants), SW480/Vector, SW480/SPON2, SW620/Scramble, SW620/shSPON2#1, SW620/shSPON2#2, MC38/Vector, MC38/SPON2, MC38/Scramble, MC38/shSpon2#1 and MC38/shSpon2#2 cells (5 × 10^6^ /100 mm dish) were incubated for 24 h. Media were removed and replaced with 8 ml serum-free L-15 or RPMI 1640. Supernatants were collected 24 h later with any floating cells removed by 0.45 mm filter. The cell suspensions collected in ultrafiltration centrifuge tube were centrifuged for 10 min, and the collected supernatants were stored at − 80℃ until further use.

### ELISA

The amounts of IL10, CCL2, and CSF1 protein in the supernatant were respectively determined using human IL10, CCL2, CSF1 specific ELISA kits (DAKEWE, 1,111,002; MEIMIAN, MM-2022H2; MEIMIAN, MM-0081H2). All experiments were performed according to the manufacturer’s instructions.

### Transendothelial migration assay (iTEM)

HUVEC monolayers or C166 cells on Transwell inserts were treated with F-12 K or DMEM media containing 10% FBS for 4 h at 37 °C. Inserts were placed over 24-well plates coated with a thin layer of Matrigel (354,262, Corning). DIL-labeled THP-1 cells were added to the top chamber with HUVECs and allowed to transmigrate through HUVECs. DIL-labeled mouse M0 macrophages were added to the top chamber with C166 and allowed to transmigrate through C166 cells. After 48 h of migration, the top chamber was removed and the cell number in the bottom chamber was fixed and stained. Transwell inserts were counterstained with DAPI and observed by fluorescence microscopy. The results were quantified by counting the number of THP-1 cells and mouse M0 macrophages passing through the endothelium in the same field (20 ×) and expressed as standardized values for at least three separate experiments. The quantification of the assay was carried out in at least three separate experiments, with each Transwell counting 5 fields.

### Cell adhesion assay

HUVECs (2 × 10^5^) were cultured on coverslips. Prior to the cell adhesion assay, green fluorescent protein (GFP)-labeled HUVECs were pretreated with conditioned medium for 2 h. Then, DIL-labeled THP-1 cells were cocultured with THP-1 cells for 2 h. Subsequently, the cultured cells were gently washed 3 times with PBS to remove the non-adhered THP-1 cells. The DIL-labeled THP-1 cells adhered to the HUVEC monolayer from ten random fields were counted under a fluorescence microscope.

### Immunofluorescence staining

For immunofluorescence staining, 4 μm sections were cut from the paraffin-embedded blocks. Antigen retrieval was performed in a pressure cooker (95 °C for 30 min). Slices were blocked with PBS containing bovine serum albumin at room temperature for 1 h. The slides were placed in primary antibodies and incubated at 4 °C overnight. Primary antibodies against PYK2 (bs-3357R, Bioss), Zyxin (60,254–1-Ig, Proteintech), Mac2 (125,402, BioLegend), and ZO-1 (66,452–1-Ig, Proteintech) were used. Then, the slides were incubated with a mixture of two secondary antibodies for 1 h in a dark room. The following secondary antibodies were used: Alexa Fluor 488-labeled anti-rabbit (A23220, Abbkine), Alexa Fluor 488-labeled anti-rat (A23240, Abbkine), Alexa Fluor 594-labeled anti-rat (A23440, Abbkine), Alexa Fluor 488-labeled anti-mouse (A23210, Abbkine), Alexa Fluor 594-labeled anti-mouse (A23410, Abbkine), and DyLight488 Phalloidin (12935S, Cell Signaling Technology). Slides were counterstained with DAPI and observed by fluorescence microscopy.

### Migration assay

An 8-μm-pore filter membrane of the Boyden chamber was used for the migration assay. RAW264.7 cells (1 × 10^5^) were seeded in the upper chamber, and 300 μL of conditioned medium was added to the lower chamber as an inducer. After incubation for 36 h, the filter was removed and fixed with neutral formaldehyde and then stained with hematoxylin. Three independent experiments were performed.

### Tumor models and treatments

Female C57 BL/6 mice (4–6 weeks old) obtained from the Animal Center of Southern Medical University, Guangzhou, China, were injected in the right flank with MC38/Vector, MC38/SPON2, MC38/Scramble, MC38/shSpon2#1 and MC38/shSpon2#2 cells (1 × 10^6^ cells/mouse) on day 0. A CSF1R-specific inhibitor (BLZ945) (T6119, TOPSCIENCE) and DMSO control were administered on days 6, 7, 8, 9, 10, 11 and 12 after tumor inoculation through intragastric administration at a dosage of 200 mg/kg. When the average volume of tumors was over 200 mm^3^, anti-mouse IL10 neutralizing monoclonal antibodies (504,908, Biolegend; 500ug/kg, qd), anti-mouse integrin β1 neutralizing monoclonal antibodies (102,202, Biolegend; 1 mg/kg, qd), defactinib (T1996, TOPSCIENCE; 10 mg/kg, qd) or normal saline (NS) (equal volume/qd, control) were injected intraperitoneally daily for one week respectively. Tumors were measured every two days. Mice were generally sacrificed when tumors became necrotic or their volume reached 2500 mm^3^, which was recorded as death for the tumor growth curve. Surgical orthotopic injection of CRC cells (1 × 10^6^ per mouse) onto the mesentery of the cecum were performed in C57BL/6 mice after anesthesia was administered. The mice were euthanized 30 days after surgery, the individual organs were excised, and metastases were observed by histological analysis.

### Flow cytometry

CRC tumor single cells were isolated using a mouse tumor dissociation kit (130–096-730, Miltenyi Biotec). Single tumor cells were stained with the indicated antibodies for 30 min on ice. Antibodies against M2-TAMs: CD45 (APC-Cy7, Cat# 103,116, BioLegend), CD11b (BV605, Cat# 101,237, BioLegend), F4/80 (BV421, Cat# 123,137, BioLegend), and CD206 (APC, Cat# 141,708, BioLegend). After two washes, the cell pellet was resuspended and analyzed by flow cytometry.

### Computational Analysis

To quantify the immune infiltration for each sample, the gene expression data of 433 TCGA Colon (COAD) and Rectum (READ) cancer samples were downloaded from GDC database, which is the normalized RSEM expression. Single sample gene set enrichment analysis (ssGSEA) was applied via the “GSVA” package based on 28 immune cell gene sets (Supplementary Table S2) that retrieved from previous study [47]. For the analysis of correlation between expression of SPON2 and expression of M2-TAM signature, the 433 TCGA colorectal samples were first clustered using M2-TAM signatures adapted as previously described (Supplementary Table S3) [48] into M2-Low, M2-Medium and M2-High (distance between pairs of samples was measured by Manhattan distance and clustering was then performed using Ward’s method hierarchical clustering). Then expression of SPON2 in each sample of each cluster was analyzed.

### Statistical analysis

SPSS 20.0 was used for all statistical analyses. Pearson correlation analysis was used for expression correlation analysis. The Kaplan–Meier method was used to analyze the survival rate. Two-tailed independent Student’s t-test was used to analyze two groups. A value of *p* < 0.05 was considered significant (* *p* < 0.05, ** *p* < 0.01, *** *p* < 0.001, **** *p* < 0.0001).

## Results

### SPON2 is positively correlated with M2-TAM infiltration and poor prognosis in CRC patients

**Fig. 1:**
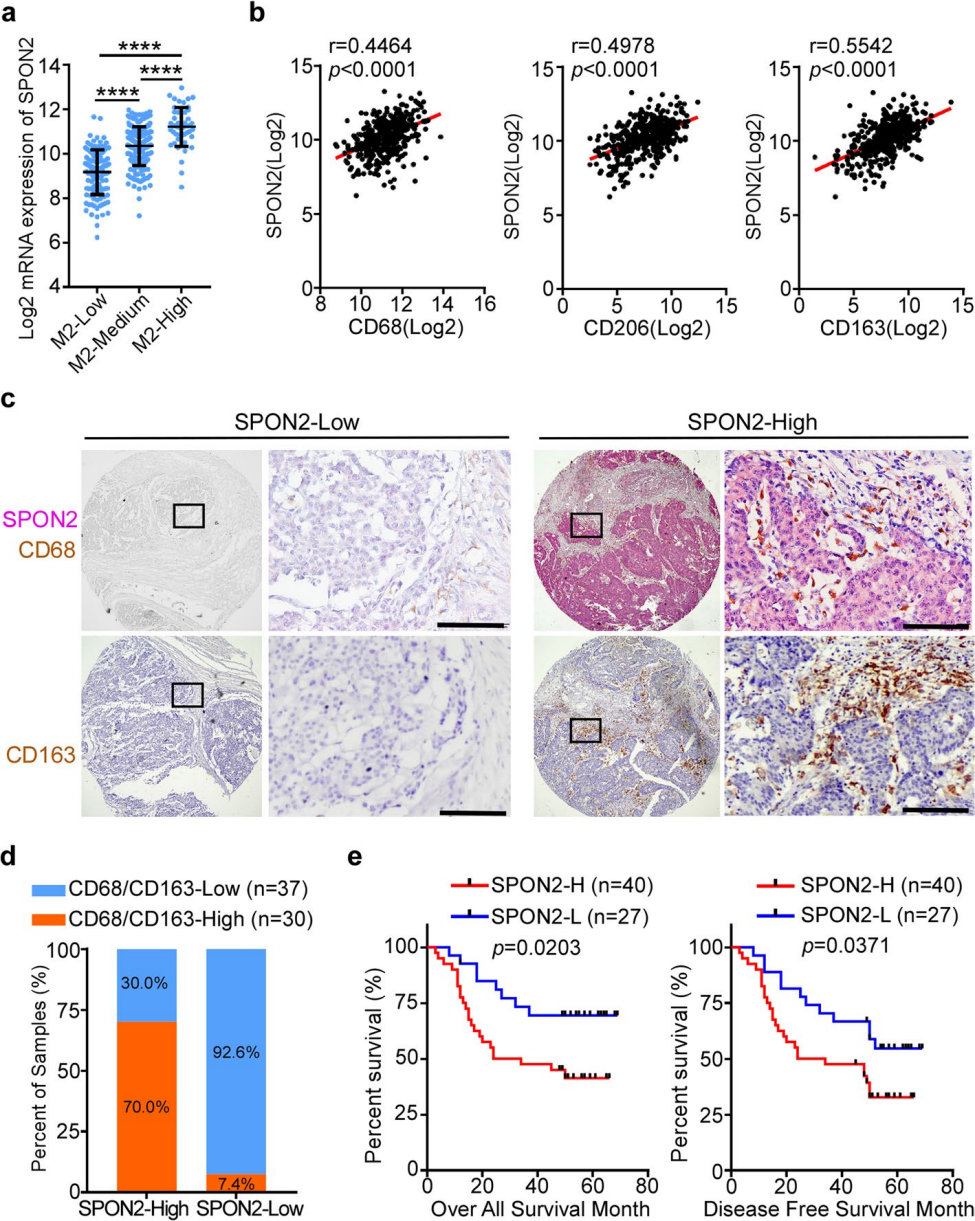
SPON2 is positively correlated with M2-TAM infiltration and poor prognosis in CRC patients. **a** The expression of SPON2 in the M2-low (*n* = 125), M2-medium (*n* = 208), and M2-high (*n* = 49) groups of TCGA-COAD and READ patients. Student’s t test, **** *p* < 0.0001. **b** The correlation between mRNA levels of SPON2 and M2-TAM markers (CD68, CD206 and CD163) of TCGA-COAD and READ patients. Two-tailed Pearson test. *n* = 458. **c** Representative double immunohistochemistry images of SPON2 and CD68 expression and representative immunohistochemistry images of CD163 in the same CRC tissue microarray (*n* = 67) (red: SPON2, brown: CD68, CD163). Scale bar, 100 μm. **d** Pearson's chi-squared test was used to analyze the correlation between SPON2 expression and CD163^+^ TAM infiltration in CRC patients (*n* = 67). χ^2^ = 25.540, *p* < 0.0001. **e** Kaplan–Meier analyses of the overall survival and disease-free survival of CRC patients with low versus high expression of SPON2 (*n* = 67)

To analyze the correlation between SPON2 expression and immune cell populations, gene expression signature analyses (GSEA) were performed using TCGA-COAD and READ data (Supplementary Figure S1a). Pearson correlation analysis showed that high SPON2 expression was significantly correlated with enrichment of suppressive immune cells, including M2-TAMs, regulatory T cells and MDSCs (Supplementary Figure S1b), especially in patients with stage IV CRC (Supplementary Figure S 1c). In addition, SPON2 expression was much higher in patients with higher expression of M2-TAM signature than those with lower expression of M2-TAM signature (Fig. 1a). Pearson correlation analysis revealed that increased SPON2 mRNA levels correlated with increased mRNA levels of M2-TAM markers, including CD68, CD206 and CD163 (Fig. 1b). To further confirm the correlation between SPON2 expression and M2-TAM infiltration, an IHC double staining analysis was performed in 67 paraffin-embedded archived CRC tissue samples using anti-SPON2, anti-CD68 and anti-CD163 antibodies. The macrophage-specific marker CD68 and M2 type-specific marker CD163 were used to distinguish M2-TAMs in human CRC tissue microarrays. The results showed that SPON2 protein was mainly localized in the cytoplasm of tumor cells and areas of ECM (Fig. 1c). In addition, SPON2 expression is significantly higher in the samples with more CD68/CD163-high TAMs (Fig. 1d). Moreover, patients with low SPON2 had longer overall survival (Fig. 1e, log-rank, *p* = 0.0203) and disease-free survival times (Fig. 1e, log-rank, *p* = 0.0371). Collectively, these data demonstrate that SPON2 expression is positively correlated with M2-TAM infiltration and CRC progression.

### SPON2 derived from CRC cells promotes TAMs migration and infiltration in tumors

**Fig. 2:**
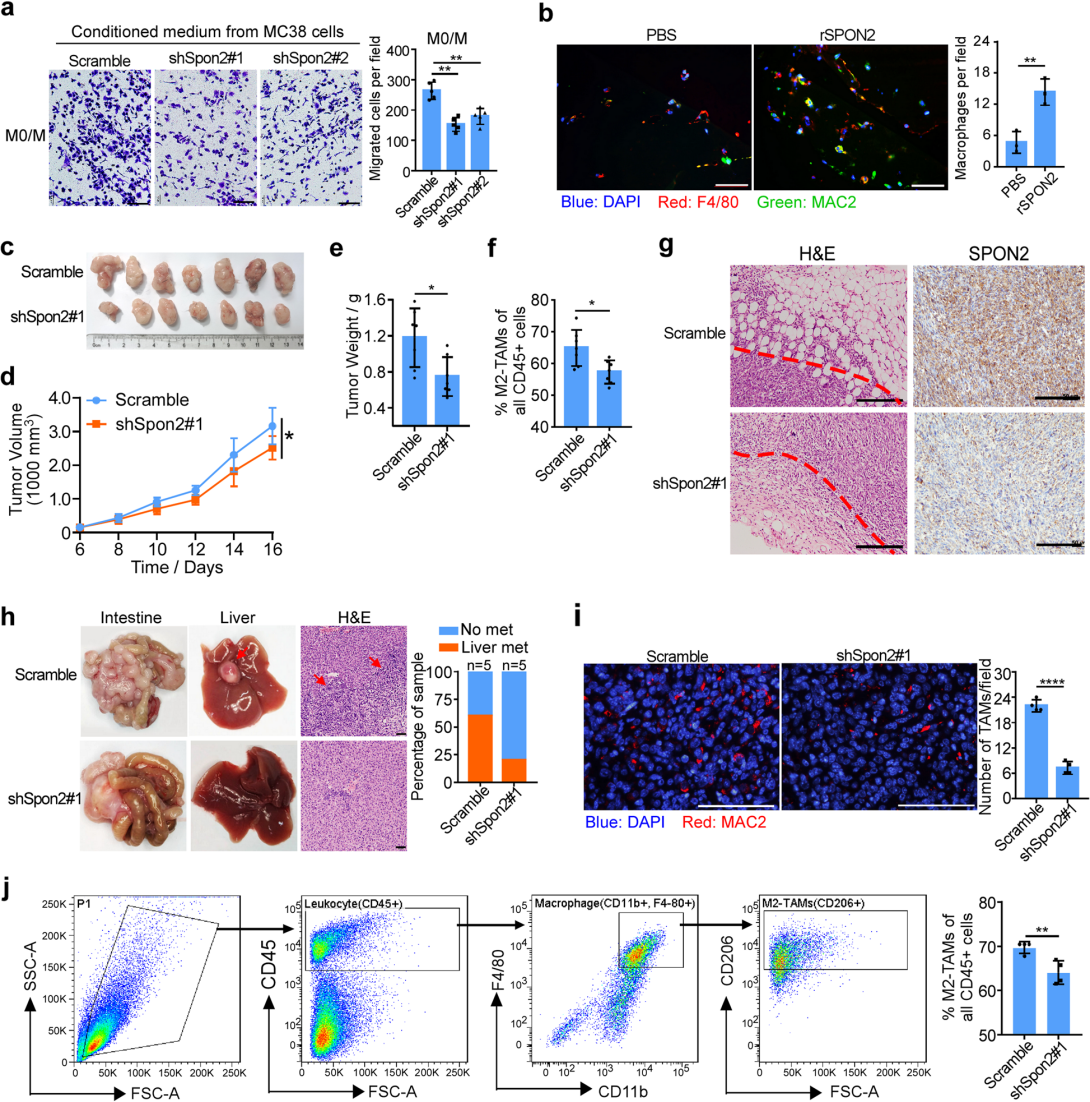
SPON2 derived from CRC cells promotes TAM migration and infiltration in tumors. **a** Migration of primary mouse macrophages (M0/M) toward conditioned medium from stable cell lines. Scale bar, 100 μm. **b** Left panel, representative immunofluorescence for MAC2 and F4/80 in growth factor-reduced Matrigel plugs supplemented with PBS or rSPON2 (1 mg/mL) and subcutaneously injected into C57 BL/6 mice. Right panel, quantification of F4/80^+^MAC2^+^ macrophages in Matrigel plugs. Scale bar, 100 μm. **c** Gross images of MC38/Scramble and MC38/shSpon2#1 subcutaneous tumors in C57 BL/6 mice. **d** Tumor growth curve of MC38/Scramble and MC38/shSpon2#1 subcutaneous tumors in C57 BL/6 mice. Two-way ANOVA test, *p < 0.05. **e** Weights of MC38/Scramble and MC38/shSpon2#1 subcutaneous tumors. Student’s t test, *p < 0.05. **f** Flow cytometric quantification showing the decreased infiltration of M2-TAMs (CD45^+^/CD11b^+^/F4/80^+^/CD206^+^). Student’s t test, *p < 0.05. **g** Representative images of MC38/Scramble and MC38/shSpon2#1 subcutaneous tumors. Hematoxylin and eosin (H&E) staining shows the histology of tumor tissues. **h** The representative gross images of the intestines and livers from MC38/Scramble and MC38/ shSpon2#1 orthotopic tumors. Sections of the liver were stained with H&E. Scale bar, 50 μm; the right graph shows quantification of no metastatic sample and liver metastatic sample. **i** Immunofluorescence (left panel) and quantification (right panel) of the macrophage marker MAC2 in MC38/Scramble and MC38/ shSpon2#1 orthotopic tumors. Scale bar, 100 μm. **j** Flow cytometric quantification showing the infiltration of M2-TAMs in orthotopic tumors. Data represent mean ± SD, ** *p* < 0.01, *** *p* < 0.001, **** *p* < 0.0001

Western Blot showed that SPON2 protein was differently expressed in CRC cell lines. The expression of SPON2 in total cell lysate was relatively high in SW620, HCT116, DLD1, RKO, and mouse CMT93 cells, while it was relatively low in SW480, CT26, and MC38 cells. (Supplementary Figure S2a). To assess the impact of SPON2 on the migration of macrophages, we established stable cell lines overexpressing-SPON2 or knockdown SPON2 using a specific shRNA (Supplementary Figure S2b-c). In vitro migration using Transwell assays showed that knockdown of SPON2 obviously decreased the migration of M0/M (Fig. 2a) and RAW264.7 cells (Supplementary Figure S2d) toward the conditioned medium. These results suggested that SPON2 promotes the migration of macrophages in vitro.

To confirm the chemoattractant ability of SPON2 in vivo, matrigel plugs with or without recombinant mouse SPON2 (rSPON2) were implanted subcutaneously into C57 BL/6 mice, showing that SPON2 supplementation significantly increased F4/80^+^ MAC2^+^ macrophage density in the matrigel plugs (Fig. 2b). Subcutaneous injection of MC38 cells showed that knockdown of SPON2 inhibited tumor growth (Fig. 2c-e), the infiltration of M2-TAMs (Fig. 2f) and tumor invasion (Fig. 2g). In addition, orthotopic injection of stable cell lines into the mesentery of the cecum was performed. The data showed that knockdown of SPON2 decreased the liver metastasis of orthotopic tumor and macrophages infiltration (Fig. 2h and i). Flow cytometry further confirmed the decreased M2-TAM (CD45^+^/CD11b^+^/F4/80^+^/CD206^+^) infiltration in SPON2-knockdown tumors (Fig. 2j). In addition, knockdown of SPON2 decreased the MDSC (CD45^+^/CD11b^+^/F4/80^−^/Gr-1^+^) and Treg (CD45^+^/CD3e^+^/CD4^+^/FoxP3^+^) infiltration in tumors (Supplementary Figure S2e-f). These results suggested that SPON2 promotes tumor progression and infiltration of suppressive immune cells infiltration, including M2-TAMs, MDSCs, and Tregs.

### SPON2 promotes transendothelial migration of monocytes

**Fig. 3:**
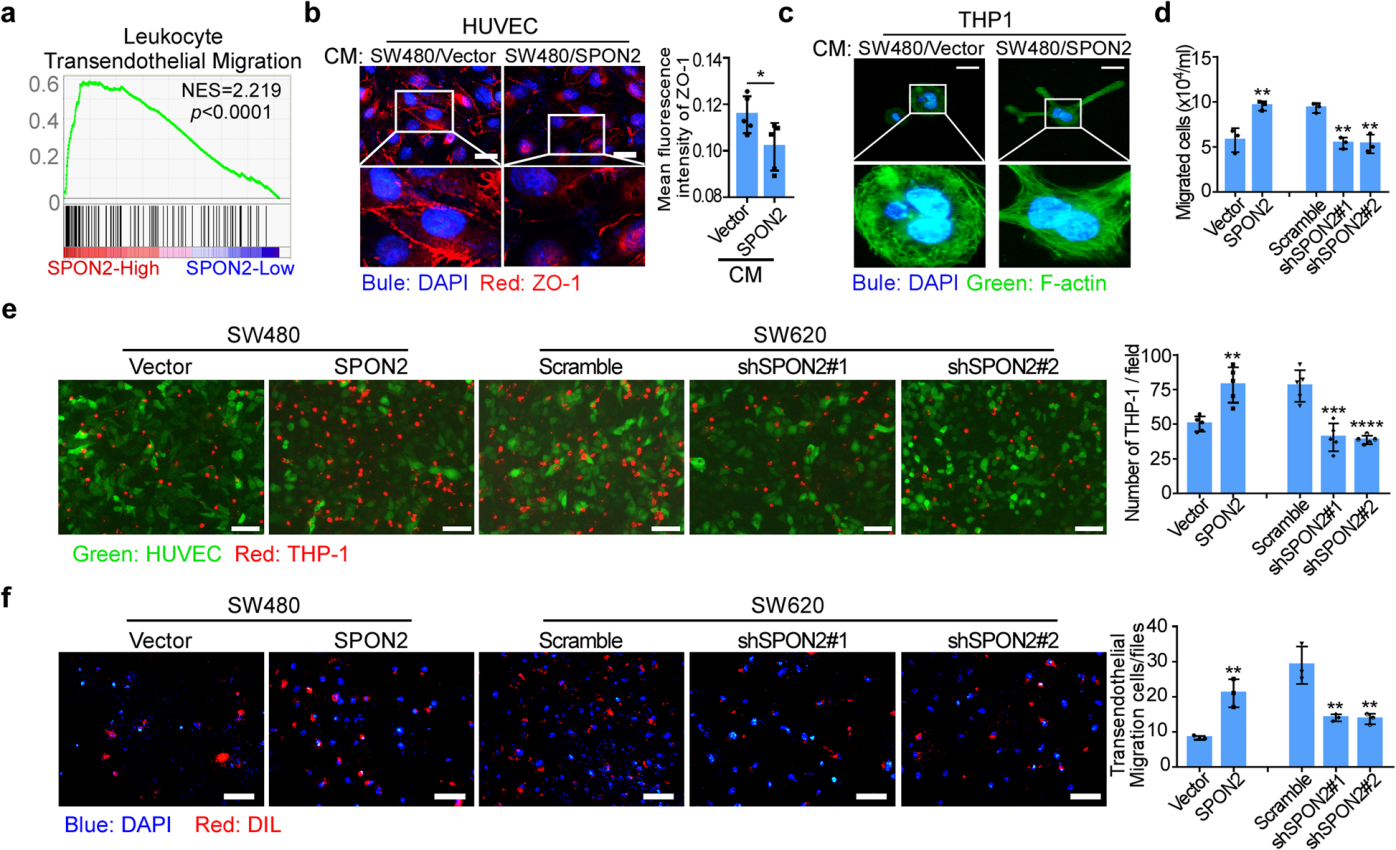
SPON2 promotes TAM transendothelial migration. **a** GSEA identified the leukocyte transendothelial migration signature as the top activated pathway in SPON2-high CRC patients (TCGA COAD). **b** Representative immunofluorescence for ZO-1 in HUVECs cocultured with THP-1 and treated with conditioned media (CM) from SW480/Vector and SW480/SPON2. Scale bar, 50 μm. **c** Immunofluorescence of F-actin (green) in THP-1 cells pretreated with conditioned medium (CM) from stable cell lines (SW480/Vector and SW480/SPON2). Nuclei were counterstained with DAPI (blue). Scale bar, 20 μm. **d** Migration of THP-1 s toward conditioned medium from stable cell lines (SW480/Vector, SW480/SPON2, SW620/ Scramble, SW620/shSPON2#1 and SW620/shSPON2#2). **e** THP-1 s were stained with DIL (red) before pretreatment with conditioned medium as indicated and were cocultured with HUVECs labeled with pCDH-GFP (green) for 2 h. Cells were observed under a fluorescence microscope and counted from 5 random fields. Scale bar, 100 μm. **f** Transendothelial migration of THP-1 s toward conditioned medium from stable cell lines. Scale bar, 100 μm. Student’s t test, * *p* < 0.05, ** *p* < 0.01, *** *p* < 0.001, **** *p* < 0.0001

TAMs are classically thought to be derived from monocytic precursors circulating in blood and recruited to the tumor site by several cytokines [25]. Gene Set Enrichment Analysis (GSEA) using the TCGA COAD dataset showed that high SPON2 expression was positively correlated with enrichment of the leukocyte transendothelial migration signaling pathway signature (Fig. 3a). To test whether SPON2 affect the transendothelial step of recruitment of monocytes into the tumor site, HUVECs were cultured with conditioned medium from SPON2 overexpression cultures. Notably, the cell membrane tight junctions, indicated by ZO-1 expression, were obviously ruptured in HUVECs added with conditioned medium from SPON2 overexpression cultures (Fig. 3b). Stress fibers and focal adhesions are complex protein arrays that produce, transmit and sense mechanical tension, which can produce protrusive forces using energy of actin polymerization and contractile forces [21]. We then examined whether SPON2 could promote migration of monocytes by observing the formation of stress frbres in THP-1 cells. F-actin staining showed more stress fiber actin bundles in THP-1 cells cultured with conditioned medium from SPON2 overexpression cultures than in the control group, which indicated morphological changes and enhanced migration ability of THP-1 (Fig. 3c). Moreover, overexpression of SPON2 in tumor cells significantly increased, while knockdown of SPON2 in tumor cells decreased the migration of THP-1 cells (Fig. 3d) and the numbers of THP-1 cells adhered to the HUVECs (Fig. 3e). Furthermore, overexpression of SPON2 in tumor cells obviously increased, while knockdown of SPON2 in tumor cells dramatically decreased the transendothelial movement (Fig. 3f). Taken together, these results indicate that SPON2 promotes the transendothelial migration of monocytes.

### SPON2 activated IL10, CCL2 and CSF1 to enhance polarization to the M2 phenotype

**Fig. 4:**
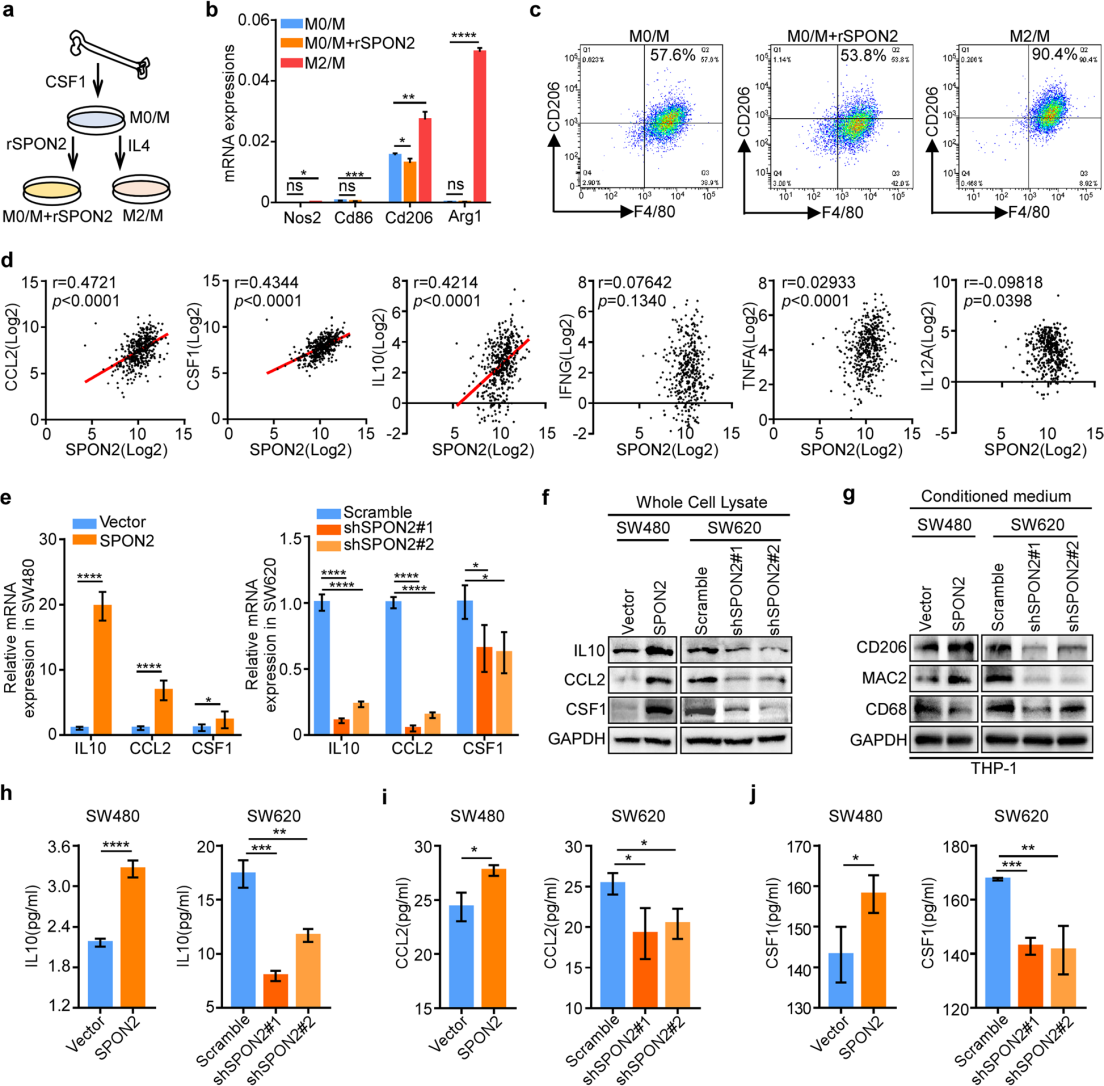
SPON2 activated IL10, CCL2 and CSF1 to enhance polarization to the M2 phenotype. **a** Schematic of the sample preparation for Q-RT-PCR and flow cytometry. **b** Q-RT-PCR showed the mRNA expression of M1 macrophage markers (Nos2 and Cd86) and M2 macrophage markers (Cd206 and Arg1). **c** Flow cytometric quantification showing the proportion of M2 macrophages (F4/80^+^, CD206^+^). **d** Correlation analysis of mRNA levels between SPON2 and cytokines that promote macrophage polarization in TCGA. Two-tailed Pearson test. *n* = 458. **e** Q-RT-PCR showed the mRNA expression of IL10, CCL2 and CSF1 in CRC cell lines. **f** Western blot analysis of IL10, CCL2 and CSF1 protein levels in the cell lysates of SW480/Vector, SW480/SPON2, SW620/Scramble, SW620/shSPON2#1 and SW620/shSPON2#2 cells. **g** Western blot analysis of CD206, MAC2 and CD68 protein levels in THP-1 cells cocultured with SW480/Vector, SW480/SPON2, SW620/Scramble, SW620/shSPON2#1 and SW620/shSPON2#2 cells. **h-j** ELISA analysis of IL10, CCL2 and CSF1 in SW480/Vector, SW480/SPON2, SW620/Scramble, SW620/shSPON2#1 and SW620/shSPON2#2 cells. In **b**, **e**, and **h-j**, data represent mean ± SD, Student’s t test, * *p* < 0.05, ** *p* < 0.01, *** *p* < 0.001, **** *p* < 0.0001

Since the high expression of SPON2 is correlated with M2-TAM infiltration in CRC tumors, we sort to test whether SPON2 could affect the M2 polarization of TAMs. Bone marrow-derived macrophages were isolated from C57 BL/6 mice and were polarized using colony-stimulating factor (CSF)-1 plus interleukin (IL)-4 (M2/M—polarized) versus CSF1 plus rSPON2 (M0/M—rSPON2) (Fig. 4a). Q-RT-PCR and flow cytometry showed that addition of rSPON2 had no effect on the expression of M2 markers or the proportion of M2 macrophages (Fig. 4b-c), suggesting that SPON2 may not directly promote M2 polarization. However, flow cytometry analysis of macrophages co-cultured with tumor cells (MC38/Scramble, MC38/shSpon2#1 and MC38/shSpon2#2) showed that knockdown SPON2 significantly decreased the polarization toward M2-like macrophages (Supplementary Figure S3).​ TCGA-COAD data showed that the mRNA expression of SPON2 was positively correlated with the mRNA expression of M2 polarization factors such as CCL2, CSF1 and IL10 but not with M1 polarization factors, such as TNF-α, IFN-γ and IL12 (Fig. 4d). In addition, overexpression of SPON2 enhanced, while knockdown of SPON2 reduced IL10, CCL2 and CSF1 expression levels (Fig. 4e-f) and secretion (Fig. 4h-j) in CRC tumor cells. Moreover, the expression of macrophages markers (CD68 and MAC2) and M2-like macrophages markers (CD206) significantly increased in THP-1 cells co-cultured with SPON2-overexpressing CRC tumor cells compared with the control tumor cells (Fig. 4g). In contrast, the expression of CD206, CD68 and MAC2 were significantly decreased in the THP-1 cells co-cultured with SPON2-knockdown CRC cells compared with the control group (Fig. 4g). These results suggested that SPON2 may indirectly induce M2-polarization though upregulating cytokines including IL10, CCL2 and CSF1 expression in tumor cells.

### Blocking M2 polarization and Macrophage depletion inhibited the SPON2-induced tumors growth and invasion

**Fig. 5:**
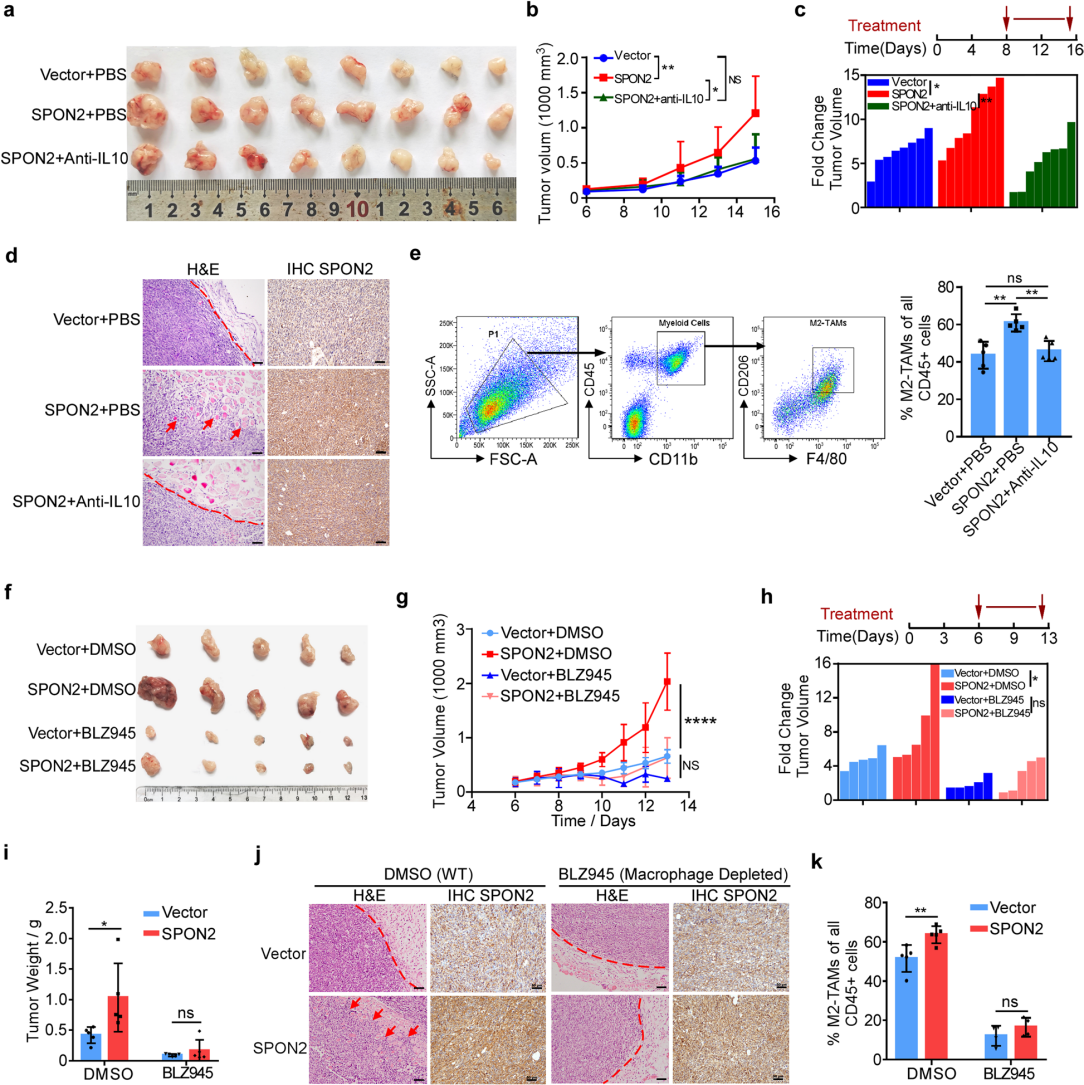
Blocking M2 polarization of TAMs and Macrophage depletion inhibited the SPON2-induced tumors growth and invasion. **a** Gross images of MC38/Vector + PBS, MC38/SPON2 + PBS and MC38/SPON2 + IL10 subcutaneous tumor. **b** Growth curve in the different treatment groups. Two-way ANOVA test, *p < 0.05, **p < 0.01. **c** Anti-IL10 neutralizing antibody treatment time diagram and fold change of tumor volume in each case (final volume / initiate volume). **d** Hematoxylin and eosin (H&E) staining shows the histology of subcutaneous tumor tissues. IHC shows tumor cells with SPON2 expression. The arrows indicated the tumor invasion. Scale bar, 50 μm. **e** Flow cytometric quantification showing percentages M2-TAMs of all CD45 + cells in subcutaneous tumors, **p < 0.01. **f** Gross images of MC38/Vector and MC38/SPON2 subcutaneous tumors in C57BL/6 mice treated with BLZ945 or isotope control (DMSO). **g** Tumor growth curve in the different treatment groups. Two-way ANOVA test, ****p < 0.0001. **h** BLZ945 treatment time diagram and fold change of tumor volume in each case (final volume / initiate volume). **i** Tumor weights of mice in the different treatment groups at the end point. *p < 0.05. **j** Hematoxylin and eosin (H&E) staining shows the histology of subcutaneous tumor tissues. IHC shows tumor cells with SPON2 expression. The arrows indicated the tumor invasion. Scale bars, 50 μm. **k** Flow cytometric quantification showing the effect of SPON2 on the infiltration of M2-TAMs upon BLZ945 treatment. **p < 0.01

To assess the role of SPON2 in macrophage polarization and tumor progression in vivo, MC38 cells overexpressed with SPON2 or a control vector were subcutaneously injected into C57 BL/6 mice. Mice were treated with anti-IL10 neutralizing antibody for 7 days to inhibit M2 polarization of TAMs. The results showed that blocking IL10 inhibited growth and invasion of SPON2-overexpressed MC38 tumors (Fig. 5a-d, Supplementary Figure S4a). In addition, blocking IL10 suppressed the infiltration of M2-TAMs in SPON2-overexpressed MC38 tumors (Fig. 5e). To further investigate the role of macrophages in SPON2-induced tumor progression, mice were treated with a CSF1R-specific inhibitor (BLZ945) or DMSO for 7 days to deplete macrophages. Flow cytometry showed that BLZ945 treatment successfully reduced the macrophages infiltration in tumor (Supplementary Figure S4b). Overexpression of SPON2 dramatically accelerated tumor growth and increased tumor weight, while treatment of BLZ945 abolished the effect of SPON2 on tumor growth and invasion (Fig. 5f-j). Additionally, overexpression of SPON2 significantly increase the number of tumor-infiltrated M2-TAMs in mice treated with DMSO, while no changes were observed in mice treated with BLZ945 (Fig. 5k). Taken together, these data suggest that the function of SPON2 in promoting tumor progression mainly depends on macrophages in CRC.

### SPON2 promotes cytoskeletal remodeling and by activating integrin β1/PYK2 axis

**Fig. 6:**
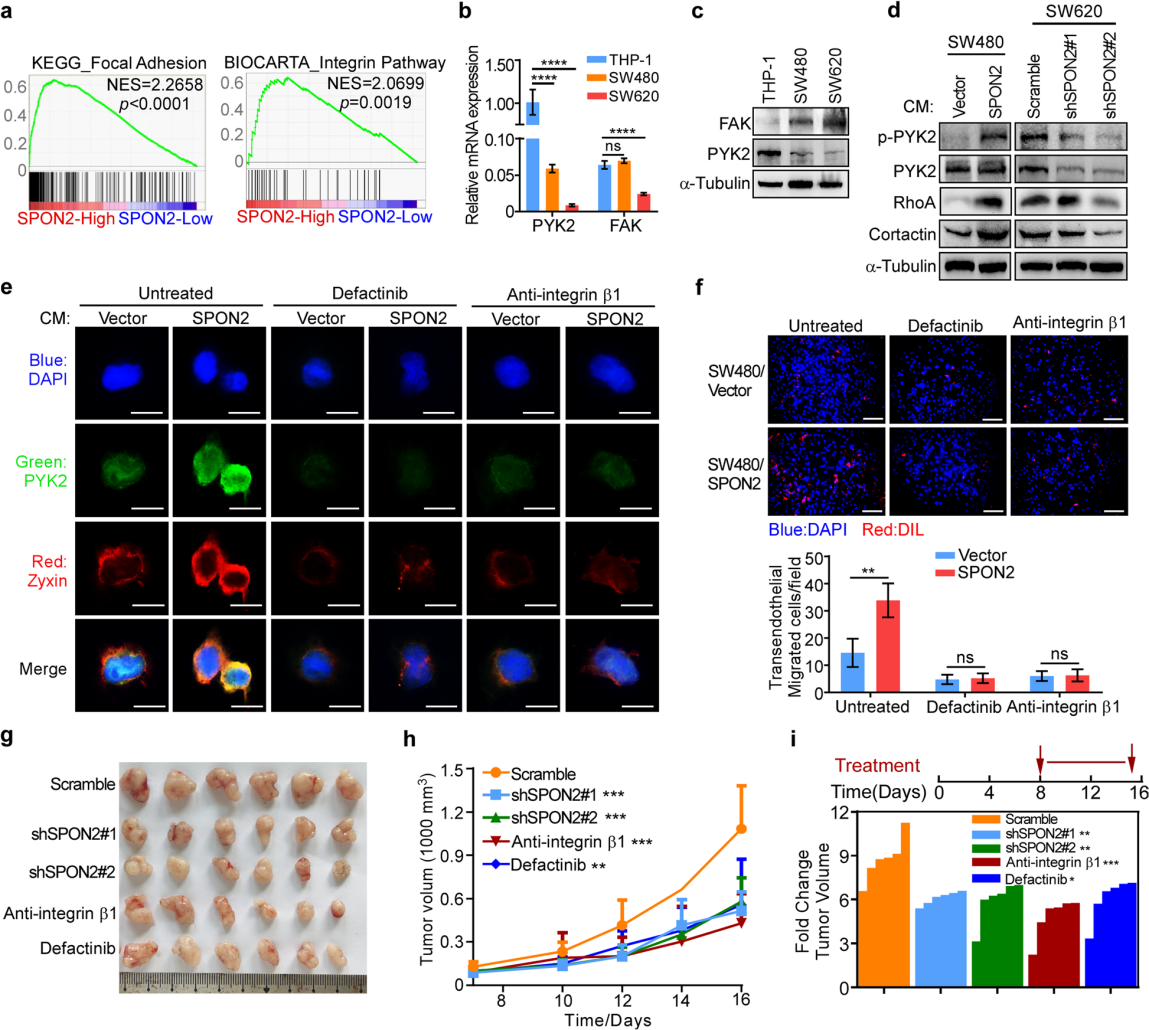
SPON2 promotes monocyte transendothelial migration and tumor growth by activating integrinβ1/PYK2 axis. **a** GSEA of the focal adhesion signature and integrin pathway signatures in the SPON2-high group compared to the SPON2-low group. NES, normalized enrichment score. **b** Q-RT-PCR showed the mRNA expression of PYK2 and FAK in CRC cell lines (SW480 and SW620) and a mononuclear cell line (THP-1). **c** Western blot analysis showed the protein expression of PYK2 and FAK in CRC cell lines (SW480 and SW620) and a mononuclear cell line (THP-1). **d** THP-1 cells were treated with conditioned medium from stable cell lines as indicated, and the expression of representative molecules in the focal adhesion pathway was analyzed by western blotting. **e** Expression and colocalization of PYK2 and zyxin in THP-1 cells with different treatments by immunofluorescence (IF) staining. Scale bars, 10 μm. **f** Transendothelial migration of THP-1 toward conditioned medium from stable cell lines with different treatments. Scale bar, 100 µm. **g** Gross images of different treatment subcutaneous tumor groups, including MC38/Scramble, MC38/shSpon2#1, MC38/shSpon2#2, anti-integrin β1 and defactinib. **h** Growth curve in the different treatment groups. Two-way ANOVA test, **p < 0.01, ***p < 0.001. **i** Treatment time diagram and fold change of tumor volume in each case (final volume / initiate volume). Data represent mean ± SD, Student’s t test, *p < 0.05, **p < 0.01, ****p < 0.0001

To explore the potential mechanism by which SPON2 induces transendothelial migration of monocytes, GSEA analysis were performed showing that the focal adhesion signature was the top pathway activated in patients with high SPON2 expression (Fig. 6a). Focal adhesion kinase (FAK) and proline-rich tyrosine kinase-2 (PYK2) are members of the FAK family, which plays a critical role in the focal adhesion pathway [49]. Q-RT-PCR and western blotting revealed high endogenous PYK2 expression in THP-1. In contrast, endogenous FAK was highly expressed in CRC cell lines but weakly expressed in THP-1 cells (Fig. 6b-c). These results suggested that PYK2 may be the major kinase that facilitates the migration of monocytes/macrophages. We then examined the effect of SPON2 on the expression of PYK2 and downstream targets. The results showed that addition of recombinant SPON2 significantly increased the expression of phospho-PYK2 and PYK2 at the protein level in RAW264.7 cells (Supplementary Figure S5a). Moreover, addition of conditioned medium from SPON2 overexpression cultures significantly increased, while addition of conditioned medium from SPON2-knockdwon cultures decreased the expression of phospho-PYK2, PYK2, RhoA and cortactin in THP-1 cells (Fig. 6d). Furthermore, ectopic SPON2 significantly increased the colocalization of PYK2 and zyxin, which is a hallmark of mature focal adhesion [50] (Fig. 6e). These data indicated that SPON2 promotes cytoskeletal remodeling by activating PYK2 in monocytes.

Previous studies have shown that SPON2 serves as α4β1 integrin a ligand in macrophages [51]. Consistently, GSEA also showed a positive correlation between SPON2 expression and the integrin pathway (Fig. 6a). To test the effect of the SPON2/integrin β1/PYK2 axis on cell migration, THP-1 cells incubated with conditioned media from SPON2 overexpression cultures were treated with the PYK2 inhibitor defactinib or anti-integrin βl neutralizing antibody. Inhibition of PYK2 or integrin β1 decreased the colocalization of PYK2 and zyxin in cells treated with SPON2 overexpression conditions (Fig. 6e). Furthermore, inhibition of PYK2 or integrin β1 obviously decreased the expression of PYK2, RhoA, and cortactin and abrogated the effect of SPON2 on increasing the expression of these proteins in cells treated with recombinant SPON2 in RAW264.7 (Supplementary Figure S5b). Moreover, addition of conditioned media from SPON2 overexpression cultures dramatically increased the migration of THP-1 cells and primary mouse macrophages (M0/M), while inhibition of PYK2 or integrin β1 abrogated the effect of SPON2 on cell transendothelial migration (Fig. 6f and Supplementary Figure S5c). To confirm the above in vitro results, defactinib or anti- integrin βl neutralizing antibody were used on MC38-bearing mice. The data showed that tumors in the scramble group were significantly larger and heavier than those in the shSpon2, anti-integrin β1 and defactinib group (Fig. 6g-i and Supplementary Figure S5d). Furthermore, blocking integrin β1 and inhibition of PYK2 replicate the effect of SPON2 suppression to cause decrease of the enrichment of M2-TAMs (Supplementary Figure S5e-f). Collectively, these results demonstrate that blocking SPON2/integrin β1/PYK2 axis impairs the transendothelial migration of monocytes and cancer-promoting functions of TAMs in vivo.

## Discussion

Dysregulation of SPON2 expression has been documented in several human cancers, including prostate cancer [42, 52], gastric cancer [53] and ovarian cancer [41]. High expression levels of SPON2 mRNA and protein predicted poor prognosis of CRC patients [54]. In the current study, we demonstrated the critical roles of the tumor-derived SPON2 in the infiltration of M2-TAMs and tumor progression in advanced CRC. High SPON2 protein levels are correlated with more infiltrated M2-TAMs and poor prognosis of patients with advanced CRC. Mechanistically, SPON2 activates the integrin-PYK2 pathway in mononuclear cells/macrophages to promote their transendothelial migration and infiltration into CRC. In contrast to our findings, SPON2 not only promotes infiltration of M1-like macrophages but also inhibits tumor cell migration and tumor metastasis in hepatocellular carcinoma [40]. The distinct effect of SPON2 on metastasis in hepatocellular carcinoma and in CRC is probably due to the discrepancy in the cell biology and TME in the two types of malignancies. For example, TAMs infiltrated in CRC frequently showed inflammatory and angiogenic features (M2-TAMs) [55]. On the contrary, SPON2 prevented F-actin assembly, thereby inhibiting hepatocellular carcinoma cell migration by α5β1 integrin inactivated RhoA [55]. Since SPON2 regulates Rho GTPase expression in DCs by interacting with the integrins α4β1 and α5β1 [56], different expression and activity of integrins in CRC and hepatocellular carcinoma may also contribute to the functional contradiction. Although integrin α5β1 is overexpressed in hepatocellular carcinoma [40], it is frequently lost in colorectal cancer cells compared with normal intestinal epithelium [57].

It has been documented that SPON2 directly binds to bacterial and viral pathogens to initiate innate immune responses and functions as an opsonin for macrophage phagocytosis [39, 58]. The SPON2/integrin β1/Rac signaling process plays a critical role in DC priming of T lymphocytes in a murine inflammation model [56]. These studies suggest an essential role of SPON2 in the function of myeloid cells during infection and inflammation processes. However, the roles of SPON2 in recruiting and function of myeloid cells in the circumstances of malignant are largely unclear. Our findings demonstrate a positive correlation between high SPON2 protein levels and greater M2-TAM infiltration in the intratumoral area in CRC patients. We also show that SPON2-driven M2-TAM infiltration plays an important role in tumor invasion and metastasis in CRC. In detail, SPON2 promotes monocytes/macrophage migration by activation PYK2 signaling in these cells. TAMs are classically thought to be derived from peripheral blood monocytes [59–61]. A multistep cascade of capture, rolling, slow rolling, firm adhesion, adhesion strengthening, and intraluminal crawling precedes the transendothelial migration of monocytes [62, 63]. Transendothelial migration is controlled by complicated signaling and requires the coordination of alterations in cell shape and adhesive properties that are mediated by cytoskeletal dynamics. FAK and PYK2 are members of the FAK family [64, 65], which plays a critical role in modulating the cell cytoskeleton and structures to regulate cell migration [66, 67]. Actin-binding proteins, such as paxillin, vinculin and zyxin, are recruited by the activated members of the FAK family and assemble the focal adhesion complex to regulate cell migration [49, 50, 68]. This is the first report identifying SPON2 promote the transendothelial migration of monocytes to maintain the infiltration of TAMs. We confirmed that SPON2-induced monocyte transendothelial migration is regulated by the focal adhesion signaling pathway. SPON2 can activate PYK2 in macrophages and monocytes by interaction with integrin β1. Activated PYK2 recruits more zyxin to promote the formation of focal adhesion complexes involved in stress fiber maintenance of migrating cells. In addition, SPON2 increases the activity and expression of RhoA and cortactin by activating PYK2, which promotes cytoskeletal remodeling and transendothelial migration.

Tumor cells secrete significant amounts of cytokines to promote M2 phenotype polarization in tumor microenvironment. IL10 is highly expressed in colorectal cancer cells, and polarizes TAMs to the M2 phenotype, which promotes cancer cell migration and metastasis.[28, 69]. Inhibition of CCL2/CCR2 or CSF1/CSF1R signaling pathway can reduce the intratumoral infiltration of M2-TAM and inhibit tumor growth and metastasis [26, 70]. Besides the effects on promoting cell migration, SPON2 also helps M2-polarization of the macrophages during their recruitment into the TME by upregulating the expression and production of cytokines, including IL10, CCL2 and CSF1. Blocking IL10 abrogates the SPON2-mediated intratumoral M2-TAM enrichment into the tumor and tumor growth. In addition, AP1, which activated by ERK1/2 pathway was reported as upstream regulator of cytokines IL10, CCL2 and CSF1 [28, 71–75]. It has been documented that SPON2 protein in the ECM interacts with integrin β1 and activates focal adhesion pathway and ERK1/2 pathway [76, 77]. Therefore, we speculated that SPON2 may activate ERK/AP1 pathways to upregulate the expression of IL10, CCL2 and CSF1 by binding integrin β1. Furthermore, it has been reported that CCL2, IL10, and CSF1 not only expressed in tumor cells, but also in macrophages [30]. It is interesting to determine whether SPON2 affects this cytokines in macrophages. Overall, we uncovered a novel role for SPON2 in the regulation of macrophage polarization in colorectal cancer. However, how SPON2 regulates cytokine expression remains to be explored.

## Conclusions

**Fig. 7:**
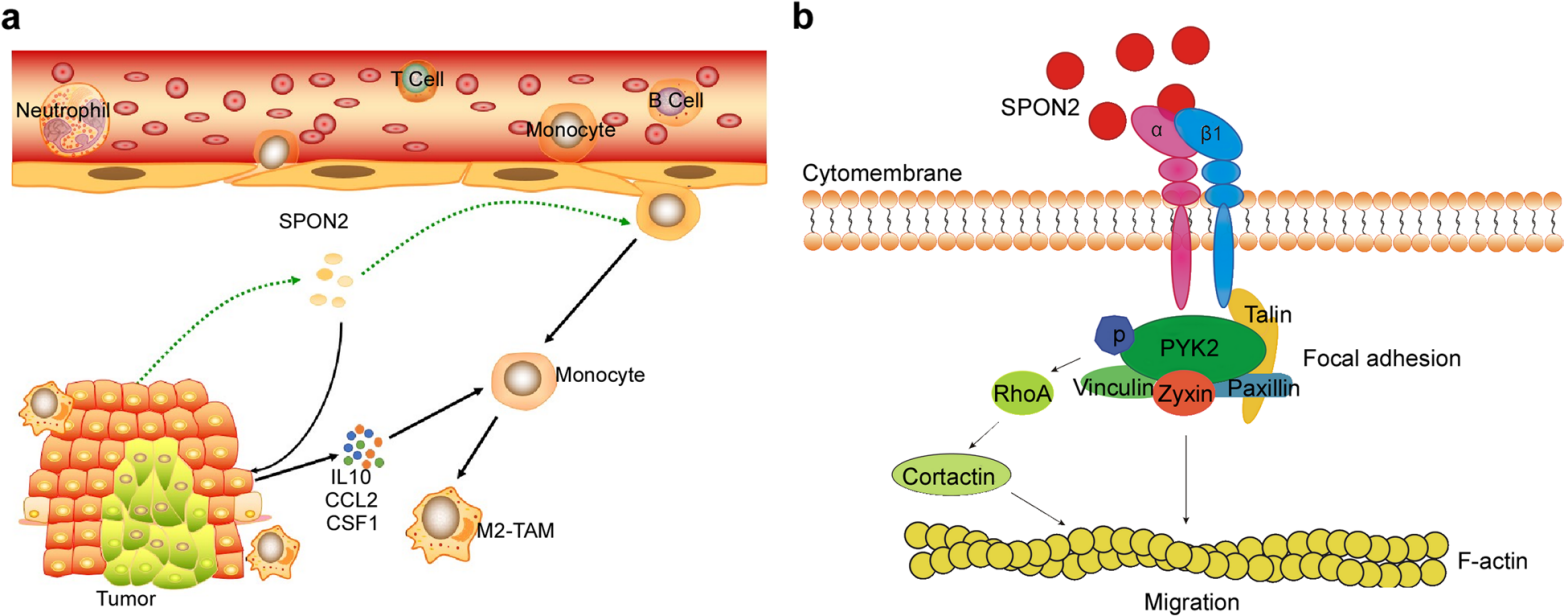
Proposed model. **a** Model of SPON2 biological function. SPON2 promotes transendothelial migration of monocytes and induces polarization of TAMs toward the M2 phenotype by stimulating gene expression of IL10, CCL2 and and CSF1 in CRC cells to maintain the infiltration of M2 tumor-associated macrophages. **b** Schematic representations of the role of the SPON2/integrin β1/PYK2 axis in the recruitment of TAMs. SPON2 activates PYK2 in macrophages and monocytes by binding integrin β1. Activated PYK2 recruits more zyxin to promote the formation of focal adhesion complexes that are involved in stress fiber maintenance of migrating cells

In conclusion, our findings demonstrate that SPON2 plays a crucial role in promoting transendothelial migration of monocytes to maintain the infiltration of TAMs and that this function is mediated by the SPON2/integrin β1/PYK2 axis. In addition, SPON2 indirectly induces polarization of TAMs toward the M2 phenotype by stimulating the expression and production of IL10, CCL2 and CSF1 in CRC cells. Moreover, SPON2-driven M2-TAMs infiltration plays an important role during CRC tumor growth and metastasis (Fig. 7). These findings suggest that SPON2 is a biomarker guiding the use of macrophage-targeting strategies and a potential therapeutic target in advanced CRC.

## Supplementary Information

**Additional file 1: Supplementary Figure S1.** Correlation between SPON2 expression and immune cell signatures in TCGA COAD and READ data. **a.** The heat map of the expression of immune cells between high- and low-expressed SPON2. **b.** The Pearson correlation between the expression of immune cells and SPON2 mRNA expression level in all sample of TCGA COAD and READ.** c.** The Pearson correlation between the expression of immune cells and SPON2 mRNA expression level in all sample with Stage IV. **Supplementary Figure S2.** SPON2 derived from CRC cells promotes TAMs migration and infiltration of MDSCs and Tregs in tumors. **a. **Western blot of SPON2 protein levels in the cell lysates and conditioned media of colorectal cancer cell lines. **b.** Western blot for the expression of SPON2 protein in whole cell lysate (WC) and conditioned medium (CM) of SW480/Vector, SW480/SPON2, SW620/Scramble, SW620/shSPON2#1 and SW620/shSPON2#2. **c.** Western blot for the expression of SPON2 protein in whole cell lysate (WC) and conditioned medium (CM) of MC38/Vector, MC38/SPON2, MC38/Scramble, MC38/shSpon2#1.and MC38/shSpon2#2. **d.** Migration of RAW264.7 cells toward conditioned medium from stable cell lines. Scale bar, 100 μm. **e.** FACS plot showing percentage of MDSCs (CD45^+^/CD11b^+^/F4/80^-^/Gr-1^+^) in orthotopic tumors from MC38/Scramble and MC38/shSpon2#1. **f.** FACS plot showing percentage of Tregs (CD45^+^/CD3e^+^/CD4^+^/FoxP3^+^) in orthotopic tumors from MC38/Scramble and MC38/shSpon2#1. **Supplementary Figure S3. **Flow cytometry analysis of the proportion of M2-like cells in M0 macrophages, M2 macrophages, and M0 macrophages co-cultured with MC38/Scramble, MC38/shSpon2#1and MC38/shSpon2#2 cell lines. **Supplementary Figure S4.** Tumor weights and infiltration of TAMs. **a.** Tumor weights of mice in the different treatment groups. **b.** Flow cytometry gating strategy for TAMs (CD45+, CD11b+, F4/80+) showing the efficiency of macrophage depletion. **Supplementary Figure S5.** SPON2 promotes monocyte transendothelial migration and tumor growth by activating integrin β1/PYK2 axis. **a.** Western blots for phospho-PYK2 and PYK2 in Raw264.7 macrophages treated with SPON2 at the indicated time points. **b.** PYK2 inhibitors (Defactinib) and integrin β1 neutralizing antibody (Anti-integrin β1) were used in Raw264.7 treated by rSPON2, and the expression of downstream molecules were analyzed by western blotting. **c. **Transendothelial migration of primary mouse macrophages M0/M toward conditioned medium from stable cell lines with different treatments. Scale bar, 100 µm. **d.** Tumor weights of mice in the different groups of treatment. **e.** Flow cytometric quantification showing the decreased infiltration of M2-TAMs in the different treatment groups. **f.** Immunofluorescence (left panel) and quantification (right panel) of MAC2 in tumors. Scale bar, 50 μm. Data represent mean ± SD, Student’s t test, * *p* < 0.05, ** *p* < 0.01, *** *p* < 0.001. **Supplementary Table S1.** Primers. **Supplementary Table S2.** Immune cell signatures. **Supplementary Table S3.** Markers of M2-TAM signature.

## Data Availability

All data generated or analyzed during this study are included in this published article and its supplementary information files.

## Abbreviations

SPON2: Spondin 2
TME: Tumor microenvironment
TAMs: Tumor-associated macrophages
ECM: Extracellular matrix
HUVEC: Human umbilical vein endothelial cells
GSEA: Gene set enrichment analysis
shRNA: Short hairpin RNA
IHC: Immunohistochemistry
H&E: Hematoxylin and eosin
BMDM: Bone marrow-derived macrophage

## References

[1] Bray F, Ferlay J, Soerjomataram I, Siegel RL, Torre LA, Jemal A (2018). Global cancer statistics 2018: GLOBOCAN estimates of incidence and mortality worldwide for 36 cancers in 185 countries. CA Cancer J Clin.

[2] Wang J, Li S, Liu Y, Zhang C, Li H, Lai B (2020). Metastatic patterns and survival outcomes in patients with stage IV colon cancer: A population-based analysis. Cancer Med.

[3] Peddareddigari VG, Wang D, Dubois RN (2010). The tumor microenvironment in colorectal carcinogenesis. Cancer Microenviron.

[4] Pollard JW (2004). Tumour-educated macrophages promote tumour progression and metastasis. Nat Rev Cancer.

[5] Grivennikov SI, Greten FR, Karin M (2010). Immunity, inflammation, and cancer. Cell.

[6] Su S, Liu Q, Chen J, Chen J, Chen F, He C (2014). A positive feedback loop between mesenchymal-like cancer cells and macrophages is essential to breast cancer metastasis. Cancer Cell.

[7] Vasiljeva O, Papazoglou A, Kruger A, Brodoefel H, Korovin M, Deussing J (2006). Tumor cell-derived and macrophage-derived cathepsin B promotes progression and lung metastasis of mammary cancer. Cancer Res.

[8] Guo Z, Song J, Hao J, Zhao H, Du X, Li E (2019). M2 macrophages promote NSCLC metastasis by upregulating CRYAB. Cell Death Dis.

[9] Zhou Q, Peng R-Q, Wu X-J, Xia Q, Hou J-H, Ding Y, et al. The density of macrophages in the invasive front is inversely correlated to liver metastasis in colon cancer. J Transl Med. 2010;8(1):1–9. 10.1186/1479-5876-8-13 PMC2841127 20141634

[10] Forssell J, Oberg A, Henriksson ML, Stenling R, Jung A, Palmqvist R (2007). High macrophage infiltration along the tumor front correlates with improved survival in colon cancer. Clin Cancer Res.

[11] Murray PJ, Allen JE, Biswas SK, Fisher EA, Gilroy DW, Goerdt S (2014). Macrophage activation and polarization: nomenclature and experimental guidelines. Immunity.

[12] Pinto ML, Rios E, Duraes C, Ribeiro R, Machado JC, Mantovani A (2019). The Two Faces of Tumor-Associated Macrophages and Their Clinical Significance in Colorectal Cancer. Front Immunol.

[13] Badawi MA, Abouelfadl DM, El-Sharkawy SL, El-Aal WE, Abbas NF (2015). Tumor-Associated Macrophage (TAM) and Angiogenesis in Human Colon Carcinoma. Open Access Maced J Med Sci.

[14] Jedinak A, Dudhgaonkar S, Sliva D (2010). Activated macrophages induce metastatic behavior of colon cancer cells. Immunobiology.

[15] Chanmee T, Ontong P, Konno K, Itano N (2014). Tumor-associated macrophages as major players in the tumor microenvironment. Cancers (Basel).

[16] Zhang Y, Sime W, Juhas M, Sjolander A (2013). Crosstalk between colon cancer cells and macrophages via inflammatory mediators and CD47 promotes tumour cell migration. Eur J Cancer.

[17] Koelzer VH, Canonica K, Dawson H, Sokol L, Karamitopoulou-Diamantis E, Lugli A (2016). Phenotyping of tumor-associated macrophages in colorectal cancer: Impact on single cell invasion (tumor budding) and clinicopathological outcome. Oncoimmunology.

[18] Lawrence T, Natoli G (2011). Transcriptional regulation of macrophage polarization: enabling diversity with identity. Nat Rev Immunol.

[19] Biswas SK, Mantovani A (2010). Macrophage plasticity and interaction with lymphocyte subsets: cancer as a paradigm. Nat Immunol.

[20] Cortez-Retamozo V, Etzrodt M, Newton A, Rauch PJ, Chudnovskiy A, Berger C (2012). Origins of tumor-associated macrophages and neutrophils. Proc Natl Acad Sci USA.

[21] Movahedi K, Laoui D, Gysemans C, Baeten M, Stangé G, Van den Bossche J (2010). Different tumor microenvironments contain functionally distinct subsets of macrophages derived from Ly6C(high) monocytes. Can Res.

[22] Shand FHW, Ueha S, Otsuji M, Koid SS, Shichino S, Tsukui T (2014). Tracking of intertissue migration reveals the origins of tumor-infiltrating monocytes. Proc Natl Acad Sci USA.

[23] Chitu V, Stanley ER (2006). Colony-stimulating factor-1 in immunity and inflammation. Curr Opin Immunol.

[24] Qian BZ, Li J, Zhang H, Kitamura T, Zhang J, Campion LR (2011). CCL2 recruits inflammatory monocytes to facilitate breast-tumour metastasis. Nature.

[25] Franklin RA, Liao W, Sarkar A, Kim MV, Bivona MR, Liu K (2014). The cellular and molecular origin of tumor-associated macrophages. Science.

[26] Mantovani A, Marchesi F, Malesci A, Laghi L, Allavena P (2017). Tumour-associated macrophages as treatment targets in oncology. Nat Rev Clin Oncol.

[27] Erreni M, Mantovani A, Allavena P (2011). Tumor-associated Macrophages (TAM) and Inflammation in Colorectal Cancer. Cancer Microenviron.

[28] Cheng Y, Zhu Y, Xu J, Yang M, Chen P, Xu W (2018). PKN2 in colon cancer cells inhibits M2 phenotype polarization of tumor-associated macrophages via regulating DUSP6-Erk1/2 pathway. Mol Cancer.

[29] Steinbrink K, Jonuleit H, Müller G, Schuler G, Knop J, Enk AH (1999). Interleukin-10-treated human dendritic cells induce a melanoma-antigen-specific anergy in CD8(+) T cells resulting in a failure to lyse tumor cells. Blood.

[30] Mannino MH, Zhu Z, Xiao H, Bai Q, Wakefield MR, Fang Y (2015). The paradoxical role of IL-10 in immunity and cancer. Cancer Lett.

[31] Emmerich J, Mumm JB, Chan IH, LaFace D, Truong H, McClanahan T (2012). IL-10 directly activates and expands tumor-resident CD8(+) T cells without de novo infiltration from secondary lymphoid organs. Can Res.

[32] Teng KY, Han J, Zhang X, Hsu SH, He S, Wani NA (2017). Blocking the CCL2-CCR2 Axis Using CCL2-Neutralizing Antibody Is an Effective Therapy for Hepatocellular Cancer in a Mouse Model. Mol Cancer Ther.

[33] Bonapace L, Coissieux MM, Wyckoff J, Mertz KD, Varga Z, Junt T (2014). Cessation of CCL2 inhibition accelerates breast cancer metastasis by promoting angiogenesis. Nature.

[34] Loberg RD, Ying C, Craig M, Day LL, Sargent E, Neeley C (2007). Targeting CCL2 with systemic delivery of neutralizing antibodies induces prostate cancer tumor regression in vivo. Cancer Res.

[35] Zhao L, Lim SY, Gordon-Weeks AN, Tapmeier TT, Im JH, Cao Y (2013). Recruitment of a myeloid cell subset (CD11b/Gr1 mid) via CCL2/CCR2 promotes the development of colorectal cancer liver metastasis. Hepatology.

[36] Ries CH, Cannarile MA, Hoves S, Benz J, Wartha K, Runza V (2014). Targeting tumor-associated macrophages with anti-CSF-1R antibody reveals a strategy for cancer therapy. Cancer Cell.

[37] Ries CH, Hoves S, Cannarile MA, Ruttinger D (2015). CSF-1/CSF-1R targeting agents in clinical development for cancer therapy. Curr Opin Pharmacol.

[38] Klar A, Baldassare M, Jessell TM (1992). F-spondin: a gene expressed at high levels in the floor plate encodes a secreted protein that promotes neural cell adhesion and neurite extension. Cell.

[39] He YW, Li H, Zhang J, Hsu CL, Lin E, Zhang N (2004). The extracellular matrix protein mindin is a pattern-recognition molecule for microbial pathogens. Nat Immunol.

[40] Zhang YL, Li Q, Yang XM, Fang F, Li J, Wang YH (2018). SPON2 Promotes M1-like Macrophage Recruitment and Inhibits Hepatocellular Carcinoma Metastasis by Distinct Integrin-Rho GTPase-Hippo Pathways. Cancer Res.

[41] Simon I, Liu Y, Krall KL, Urban N, Wolfert RL, Kim NW (2007). Evaluation of the novel serum markers B7–H4, Spondin 2, and DcR3 for diagnosis and early detection of ovarian cancer. Gynecol Oncol.

[42] Lucarelli G, Rutigliano M, Bettocchi C, Palazzo S, Vavallo A, Galleggiante V (2013). Spondin-2, a secreted extracellular matrix protein, is a novel diagnostic biomarker for prostate cancer. J Urol.

[43] Schmid F, Wang Q, Huska MR, Andrade-Navarro MA, Lemm M, Fichtner I (2016). SPON2, a newly identified target gene of MACC1, drives colorectal cancer metastasis in mice and is prognostic for colorectal cancer patient survival. Oncogene.

[44] Wang S, Qiu J, Liu L, Su C, Qi L, Huang C (2020). CREB5 promotes invasiveness and metastasis in colorectal cancer by directly activating MET. J Exp Clin Cancer Res.

[45] Bhardwaj A, Marsh WL, Nash JW, Barbacioru CC, Jones S, Frankel WL (2007). Double immunohistochemical staining with MUC4/p53 is useful in the distinction of pancreatic adenocarcinoma from chronic pancreatitis: a tissue microarray-based study. Arch Pathol Lab Med.

[46] Cui YM, Jiao HL, Ye YP, Chen CM, Wang JX, Tang N (2015). FOXC2 promotes colorectal cancer metastasis by directly targeting MET. Oncogene.

[47] Barbie DA, Tamayo P, Boehm JS, Kim SY, Moody SE, Dunn IF (2009). Systematic RNA interference reveals that oncogenic KRAS-driven cancers require TBK1. Nature.

[48] Bindea G, Mlecnik B, Tosolini M, Kirilovsky A, Waldner M, Obenauf AC (2013). Spatiotemporal dynamics of intratumoral immune cells reveal the immune landscape in human cancer. Immunity.

[49] Parsons JT (2003). Focal adhesion kinase: the first ten years. J Cell Sci.

[50] Zaidel-Bar R (2003). Early molecular events in the assembly of matrix adhesions at the leading edge of migrating cells. J Cell Sci.

[51] Jia W, Li H, He Y-W (2005). The extracellular matrix protein mindin serves as an integrin ligand and is critical for inflammatory cell recruitment. Blood.

[52] Qian X, Li C, Pang B, Xue M, Wang J, Zhou J (2012). Spondin-2 (SPON2), a more prostate-cancer-specific diagnostic biomarker. PLoS One.

[53] Lu H, Feng Y, Hu Y, Guo Y, Liu Y, Mao Q (2020). Spondin 2 promotes the proliferation, migration and invasion of gastric cancer cells. J Cell Mol Med.

[54] Zhang Q, Wang X-Q, Wang J, Cui S-J, Lou X-M, Yan B (2015). Upregulation of spondin-2 predicts poor survival of colorectal carcinoma patients. Oncotarget.

[55] Zhang L, Li Z, Skrzypczynska KM, Fang Q, Zhang W, O'Brien SA, et al. Single-Cell Analyses Inform Mechanisms of Myeloid-Targeted Therapies in Colon Cancer. Cell. 2020;181(2):442–59. 10.1016/j.cell.2020.03.048 32302573

[56] Li H, Oliver T, Jia W, He YW (2006). Efficient dendritic cell priming of T lymphocytes depends on the extracellular matrix protein mindin. EMBO J.

[57] Kuwada SK, Kuang J, Li X (2005). Integrin alpha5/beta1 expression mediates HER-2 down-regulation in colon cancer cells. J Biol Chem.

[58] Jia W, Li H, He YW (2005). The extracellular matrix protein mindin serves as an integrin ligand and is critical for inflammatory cell recruitment. Blood.

[59] Cortez-Retamozo V, Etzrodt M, Newton A, Rauch PJ, Chudnovskiy A, Berger C (2012). Origins of tumor-associated macrophages and neutrophils. Proc Natl Acad Sci U S A.

[60] Movahedi K, Laoui D, Gysemans C, Baeten M, Stange G, Van den Bossche J (2010). Different tumor microenvironments contain functionally distinct subsets of macrophages derived from Ly6C(high) monocytes. Cancer Res.

[61] Shand FH, Ueha S, Otsuji M, Koid SS, Shichino S, Tsukui T (2014). Tracking of intertissue migration reveals the origins of tumor-infiltrating monocytes. Proc Natl Acad Sci U S A.

[62] Gerhardt T, Ley K (2015). Monocyte trafficking across the vessel wall. Cardiovasc Res.

[63] Nourshargh S, Alon R (2014). Leukocyte migration into inflamed tissues. Immunity.

[64] Girault JA, Costa A, Derkinderen P, Studler JM, Toutant M (1999). FAK and PYK2/CAKbeta in the nervous system: a link between neuronal activity, plasticity and survival?. Trends Neurosci.

[65] Lev S, Moreno H, Martinez R, Canoll P, Peles E, Musacchio JM (1995). Protein tyrosine kinase PYK2 involved in Ca(2+)-induced regulation of ion channel and MAP kinase functions. Nature.

[66] Mitra SK, Hanson DA, Schlaepfer DD (2005). Focal adhesion kinase: in command and control of cell motility. Nat Rev Mol Cell Biol.

[67] Bauer MS, Baumann F, Daday C, Redondo P, Durner E, Jobst MA (2019). Structural and mechanistic insights into mechanoactivation of focal adhesion kinase. Proc Natl Acad Sci USA.

[68] Legerstee K, Geverts B, Slotman JA, Houtsmuller AB (2019). Dynamics and distribution of paxillin, vinculin, zyxin and VASP depend on focal adhesion location and orientation. Sci Rep.

[69] Zhang Y, Sime W, Juhas M, Sjölander A (2013). Crosstalk between colon cancer cells and macrophages via inflammatory mediators and CD47 promotes tumour cell migration. Eur J Cancer.

[70] Tacke F (2017). Targeting hepatic macrophages to treat liver diseases. J Hepatol.

[71] Hu X, Paik PK, Chen J, Yarilina A, Kockeritz L, Lu TT (2006). IFN-gamma suppresses IL-10 production and synergizes with TLR2 by regulating GSK3 and CREB/AP-1 proteins. Immunity.

[72] Qiang L, Yang S, Cui Y-H, He Y-Y. Keratinocyte autophagy enables the activation of keratinocytes and fibroblasts and facilitates wound healing. Autophagy. 2020:1–16. 10.1080/15548627.2020.1816342 PMC8496719 32866426

[73] Lin S-K, Kok S-H, Yeh FT-C, Kuo MY-P, Lin C-C, Wang C-C (2004). MEK/ERK and signal transducer and activator of transcription signaling pathways modulate oncostatin M-stimulated CCL2 expression in human osteoblasts through a common transcription factor. Arthritis Rheum.

[74] Qin L, Wu Y-L, Toneff MJ, Li D, Liao L, Gao X (2014). NCOA1 Directly Targets M-CSF1 Expression to Promote Breast Cancer Metastasis. Can Res.

[75] Chen C, Shang X, Cui L, Xu T, Luo J, Ba X (2008). L-selectin ligation-induced CSF-1 gene transcription is regulated by AP-1 in a c-Abl kinase-dependent manner. Hum Immunol.

[76] Zhang Y-L, Li Q, Yang X-M, Fang F, Li J, Wang Y-H (2018). SPON2 Promotes M1-like Macrophage Recruitment and Inhibits Hepatocellular Carcinoma Metastasis by Distinct Integrin-Rho GTPase-Hippo Pathways. Can Res.

[77] Lu H, Feng Y, Hu Y, Guo Y, Liu Y, Mao Q, et al. Spondin 2 promotes the proliferation, migration and invasion of gastric cancer cells. J Cell Mol Medi. 2020;24(1):98–113. 10.1111/jcmm.14618 PMC6933360 31691494

