# Supplementary material for: Tumor cell-derived SPON2 promotes M2-polarized tumor-associated macrophage infiltration and cancer progression by activating PYK2 in CRC
Source: J Exp Clin Cancer Res. 2021 Sep 28;40:304. doi: 10.1186/s13046-021-02108-0 (PMC8477524; doi:10.1186/s13046-021-02108-0)
Supplement: Supplementary file 1 — Additional file 1: Supplementary Figure S1. Correlation between SPON2 expression and immune cell signatures in TCGA COAD and READ data. a. The heat map of the expression of immune cells between high- and low-expressed SPON2. b. The Pearson correlation between the expression of immune cells and SPON2 mRNA expression level in all sample of TCGA COAD and READ. c. The Pearson correlation between the expression of immune cells and SPON2 mRNA expression level in all sample with Stage IV. Supplementary Figure S2. SPON2 derived from CRC cells promotes TAMs migration and infiltration of MDSCs and Tregs in tumors. a. Western blot of SPON2 protein levels in the cell lysates and conditioned media of colorectal cancer cell lines. b. Western blot for the expression of SPON2 protein in whole cell lysate (WC) and conditioned medium (CM) of SW480/Vector, SW480/SPON2, SW620/Scramble, SW620/shSPON2#1 and SW620/shSPON2#2. c. Western blot for the expression of SPON2 protein in whole cell lysate (WC) and conditioned medium (CM) of MC38/Vector, MC38/SPON2, MC38/Scramble, MC38/shSpon2#1.and MC38/shSpon2#2. d. Migration of RAW264.7 cells toward conditioned medium from stable cell lines. Scale bar, 100 μm. e. FACS plot showing percentage of MDSCs (CD45+/CD11b+/F4/80-/Gr-1+) in orthotopic tumors from MC38/Scramble and MC38/shSpon2#1. f. FACS plot showing percentage of Tregs (CD45+/CD3e+/CD4+/FoxP3+) in orthotopic tumors from MC38/Scramble and MC38/shSpon2#1. Supplementary Figure S3. Flow cytometry analysis of the proportion of M2-like cells in M0 macrophages, M2 macrophages, and M0 macrophages co-cultured with MC38/Scramble, MC38/shSpon2#1and MC38/shSpon2#2 cell lines. Supplementary Figure S4. Tumor weights and infiltration of TAMs. a. Tumor weights of mice in the different treatment groups. b. Flow cytometry gating strategy for TAMs (CD45+, CD11b+, F4/80+) showing the efficiency of macrophage depletion. Supplementary Figure S5. SPON2 promotes monocyte transendothelial migration and tumor gro [file 13046_2021_2108_MOESM1_ESM.zip › Supplementary materials-0823-clean.docx]

**Supplementary materials**

**Supplementary Figure legends**

**Supplementary Figure S1. Correlation between SPON2 expression and immune cell signatures in TCGA COAD and READ data.**

**a.** The heat map of the expression of immune cells between high -and low-expressed SPON2. **b.** The Pearson correlation between the expression of immune cells and SPON2 mRNA expression level in all sample of TCGA COAD and READ. **c.** The Pearson correlation between the expression of immune cells and SPON2 mRNA expression level in all sample with Stage IV.

**Supplementary Figure S2. SPON2 derived from CRC cells promotes TAMs migration and** **infiltration of MDSCs and Tregs in tumors.**

**a.** Western blot of SPON2 protein levels in the cell lysates and conditioned media of colorectal cancer cell lines. **b.** Western blot for the expression of SPON2 protein in whole cell lysate (WC) and conditioned medium (CM) of SW480/Vector, SW480/SPON2, SW620/Scramble, SW620/shSPON2#1 and SW620/shSPON2#2. **c.** Western blot for the expression of SPON2 protein in whole cell lysate (WC) and conditioned medium (CM) of MC38/Vector, MC38/SPON2, MC38/Scramble, MC38/shSpon2#1.and MC38/shSpon2#2. **d.** Migration of RAW264.7 cells toward conditioned medium from stable cell lines. Scale bar, 100 μm. **e.** FACS plot showing percentage of MDSCs (CD45^+^/CD11b^+^/F4/80^-^/Gr-1^+^) in orthotopic tumors from MC38/Scramble and MC38/shSpon2#1. **f.** FACS plot showing percentage of Tregs (CD45^+^/CD3e^+^/CD4^+^/FoxP3^+^) in orthotopic tumors from MC38/Scramble and MC38/shSpon2#1.

**Supplementary Figure S3.** Flow cytometry analysis of the proportion of M2-like cells in M0 macrophages, M2 macrophages, and M0 macrophages co-cultured with MC38/Scramble, MC38/shSpon2#1and MC38/shSpon2#2 cell lines.

**Supplementary Figure S4. Tumor weights and infiltration of TAMs.**

**a.** Tumor weights of mice in the different treatment groups. **b.** Flow cytometry gating strategy for TAMs (CD45+, CD11b+, F4/80+) showing the efficiency of macrophage depletion.

**Supplementary Figure** **S5.** **SPON2 promotes monocyte transendothelial migration and tumor growth by activating integrin β1/PYK2 axis.**

**a.** Western blots for phospho-PYK2 and PYK2 in Raw264.7 macrophages treated with SPON2 at the indicated time points. **b.** PYK2 inhibitors (Defactinib) and integrin β1 neutralizing antibody (Anti-integrin β1) were used in Raw264.7 treated by rSPON2, and the expression of downstream molecules were analyzed by western blotting. **c.** Transendothelial migration of primary mouse macrophages M0/M toward conditioned medium from stable cell lines with different treatments. Scale bar, 100 µm. **d.** Tumor weights of mice in the different groups of treatment. **e.** Flow cytometric quantification showing the decreased infiltration of M2-TAMs in the different treatment groups. **f.** Immunofluorescence (left panel) and quantification (right panel) of MAC2 in tumors. Scale bar, 50 μm. Data represent mean ± SD, Student’s t test, * p < 0.05, ** p < 0.01, *** p < 0.001.

**Supplementary Tables**

**Supplementary Table S1. Primers.**

| **Primers for plasmid constructs** | **Sequences (5'-3')** | |
| --- | --- | --- |
| shSPON2#1 | Forward: CCGGCGACTACAGCATGTGGAGGAACTC  Reverse: GAGTTCCTCCACATGCTGTAGTCGTTTTTG | |
| shSPON2#2 | Forward: CCGGATGTCTCCTTATAAGTTATTGCTCG Reverse: AGCAATAACTTATAAGGAGACATTTTTTG | |
| shSpon2#1 | Forward: CCGGCGGGCAGACTTCCACAGAGTTCTC Reverse: GAGAACTCTGTGGAAGTCTGCCCGTTTTTG | |
| shSpon2#2 | Forward: CCGGCCCAGCAAATTCATTCTACTACTCG Reverse: AGTAGTAGAATGAATTTGCTGGGTTTTTG | |
| **Primers for real-time PCR** |  | **Sequences (5'-3')** |
| SPON2 | Forward | GGAGAGTCCATCTGTTCCGC |
|  | Reverse | ATGCTGTAGTCGGAGCTATGC |
| PYK2 | Forward | GAAGTCCGATGAGATCCACTGG |
|  | Reverse | CTTCTGGCAAGTAGCGGATTT |
| FAK | Forward | GCTTACCTTGACCCCAACTTG |
|  | Reverse | ACGTTCCATACCAGTACCCAG |
| IL10 | Forward | ACCAAGACCCAGACATCAA |
|  | Reverse | CATTCTTCACCTGCTCCAC |
| CCL2 | Forward | TTTTCCCCTAGCTTTCCC |
|  | Reverse | GCAATTTCCCCAAGTCTCT |
| CSF1 | Forward | CAGGAGGAGCCCCAGAG |
|  | Reverse | CAGTGCGTGAGCCAATGT |
| nos2 | Forward | GCCCAGGAGGAGAGAGAT |
|  | Reverse | GCAAAGAGGACTGTGGCT |
| Cd86 | Forward | GTCACAAGAAGCCGAATCA |
|  | Reverse | GGGGTTCAAGTTCCTTCAG |
| Cd206 | Forward | CAAGCGATGTGCCTACC |
|  | Reverse | AATGCTGTGGATACTTGCC |
| Arg1 | Forward | ACGGTCTGTGGGGAAAG |
|  | Reverse | TCAGGGGAGTGTTGATGTC |

**Supplementary Table S2. Immune cell signatures.**

| **Immune cell gene signature** | | | | | |
| --- | --- | --- | --- | --- | --- |
| **Symbol** | **CellType** | **Symbol** | **CellType** | **Symbol** | **CellType** |
| ABCB4 | B.cells.naive | KLRC3 | NK.cells.resting | P2RY2 | Eosinophils |
| ADAM28 | B.cells.naive | KLRF1 | NK.cells.resting | PDE6C | Eosinophils |
| BACH2 | B.cells.naive | NAALADL1 | NK.cells.resting | PKD2L2 | Eosinophils |
| BCL7A | B.cells.naive | S1PR5 | NK.cells.resting | RGS1 | Eosinophils |
| BEND5 | B.cells.naive | TEP1 | NK.cells.resting | RNASE2 | Eosinophils |
| BRAF | B.cells.naive | TTC38 | NK.cells.resting | RRP12 | Eosinophils |
| CD22 | B.cells.naive | ZNF135 | NK.cells.resting | SAMSN1 | Eosinophils |
| CD72 | B.cells.naive | CCND2 | NK.cells.activated | SMPD3 | Eosinophils |
| CR2 | B.cells.naive | CDK6 | NK.cells.activated | SMPDL3B | Eosinophils |
| GPR18 | B.cells.naive | CTSW | NK.cells.activated | TRPM6 | Eosinophils |
| HHEX | B.cells.naive | GZMA | NK.cells.activated | ZNF165 | Eosinophils |
| IL4R | B.cells.naive | IL12RB2 | NK.cells.activated | ZNF222 | Eosinophils |
| ZNF263 | B.cells.naive | KIR2DL1 | NK.cells.activated | AQP9 | Neutrophils |
| MEP1A | B.cells.naive | KIR2DL4 | NK.cells.activated | BTNL8 | Neutrophils |
| NIPSNAP3B | B.cells.naive | KIR2DS4 | NK.cells.activated | C5AR1 | Neutrophils |
| SLC12A1 | B.cells.naive | KIR3DL2 | NK.cells.activated | CDA | Neutrophils |
| TCL1A | B.cells.naive | NCR3 | NK.cells.activated | CEACAM3 | Neutrophils |
| ZNF286A | B.cells.naive | TNFSF14 | NK.cells.activated | CREB5 | Neutrophils |
| BLK | B.cells.memory | ASGR1 | Monocytes | CSF3R | Neutrophils |
| IL7 | B.cells.memory | ASGR2 | Monocytes | CXCR1 | Neutrophils |
| NPIPB15 | B.cells.memory | CCR2 | Monocytes | CXCR2 | Neutrophils |
| SP140 | B.cells.memory | CD1D | Monocytes | FAM212B | Neutrophils |
| TRAF4 | B.cells.memory | CD33 | Monocytes | FAM65B | Neutrophils |
| ABCB9 | Plasma.cells | CFP | Monocytes | FCGR3B | Neutrophils |
| AMPD1 | Plasma.cells | FCN1 | Monocytes | FFAR2 | Neutrophils |
| ANGPT4 | Plasma.cells | UPK3A | Monocytes | FPR1 | Neutrophils |
| ATXN8OS | Plasma.cells | BHLHE41 | Macrophages.M0 | FPR2 | Neutrophils |
| C11orf80 | Plasma.cells | CHI3L1 | Macrophages.M0 | HAL | Neutrophils |
| CCR10 | Plasma.cells | COL8A2 | Macrophages.M0 | HSPA6 | Neutrophils |
| DENND5B | Plasma.cells | CSF1 | Macrophages.M0 | LST1 | Neutrophils |
| EAF2 | Plasma.cells | CXCL5 | Macrophages.M0 | MAK | Neutrophils |
| GUSBP11 | Plasma.cells | CYP27A1 | Macrophages.M0 | MEFV | Neutrophils |
| HIST1H2AE | Plasma.cells | DCSTAMP | Macrophages.M0 | MGAM | Neutrophils |
| HIST1H2BG | Plasma.cells | GPC4 | Macrophages.M0 | MMP25 | Neutrophils |
| IGHD | Plasma.cells | MARCO | Macrophages.M0 | MNDA | Neutrophils |
| IGHE | Plasma.cells | MMP9 | Macrophages.M0 | MXD1 | Neutrophils |
| IGLL3P | Plasma.cells | PLA2G7 | Macrophages.M0 | NFE2 | Neutrophils |
| KCNG2 | Plasma.cells | PPBP | Macrophages.M0 | P2RY13 | Neutrophils |
| LOC100130100 | Plasma.cells | ACHE | Macrophages.M1 | PGLYRP1 | Neutrophils |
| MAN1A1 | Plasma.cells | ADAMDEC1 | Macrophages.M1 | REPS2 | Neutrophils |
| MANEA | Plasma.cells | APOL3 | Macrophages.M1 | STEAP4 | Neutrophils |
| MAST1 | Plasma.cells | APOL6 | Macrophages.M1 | TNFRSF10C | Neutrophils |
| MROH7 | Plasma.cells | ARRB1 | Macrophages.M1 | TREM1 | Neutrophils |
| MZB1 | Plasma.cells | CCL19 | Macrophages.M1 | TREML2 | Neutrophils |
| PAX7 | Plasma.cells | CCL8 | Macrophages.M1 | VNN2 | Neutrophils |
| PDK1 | Plasma.cells | CD40 | Macrophages.M1 | VNN3 | Neutrophils |
| RASGRP3 | Plasma.cells | CXCL10 | Macrophages.M1 | ACVRL1 | Endothelial cells |
| REN | Plasma.cells | CXCL11 | Macrophages.M1 | APLN | Endothelial cells |
| SPAG4 | Plasma.cells | CXCL9 | Macrophages.M1 | BCL6B | Endothelial cells |
| ST6GALNAC4 | Plasma.cells | CYP27B1 | Macrophages.M1 | BMP6 | Endothelial cells |
| TGM5 | Plasma.cells | EBI3 | Macrophages.M1 | BMX | Endothelial cells |
| TNFRSF17 | Plasma.cells | HESX1 | Macrophages.M1 | CDH5 | Endothelial cells |
| UGT2B17 | Plasma.cells | MACF1 | Macrophages.M1 | CLEC14A | Endothelial cells |
| CD8A | T.cells.CD8 | NOD2 | Macrophages.M1 | CXorf36 | Endothelial cells |
| CD8B | T.cells.CD8 | PLA1A | Macrophages.M1 | EDN1 | Endothelial cells |
| CRTAM | T.cells.CD8 | SIGLEC1 | Macrophages.M1 | ELTD1 | Endothelial cells |
| TRAV12-2 | T.cells.CD8 | SLAMF1 | Macrophages.M1 | EMCN | Endothelial cells |
| ANKRD55 | T.cells.CD4.naive | SLC2A6 | Macrophages.M1 | ESAM | Endothelial cells |
| ATHL1 | T.cells.CD4.naive | SOCS1 | Macrophages.M1 | ESM1 | Endothelial cells |
| DSC1 | T.cells.CD4.naive | TLR7 | Macrophages.M1 | HECW2 | Endothelial cells |
| EPHA1 | T.cells.CD4.naive | TNFAIP6 | Macrophages.M1 | HHIP | Endothelial cells |
| FLT3LG | T.cells.CD4.naive | TNIP3 | Macrophages.M1 | KDR | Endothelial cells |
| GAL3ST4 | T.cells.CD4.naive | CCL14 | Macrophages.M2 | MMRN1 | Endothelial cells |
| GALR1 | T.cells.CD4.naive | CCL18 | Macrophages.M2 | MMRN2 | Endothelial cells |
| LEF1 | T.cells.CD4.naive | CCL23 | Macrophages.M2 | MYCT1 | Endothelial cells |
| MAP4K2 | T.cells.CD4.naive | CD4 | Macrophages.M2 | PALMD | Endothelial cells |
| UBASH3A | T.cells.CD4.naive | CD68 | Macrophages.M2 | PEAR1 | Endothelial cells |
| WNT7A | T.cells.CD4.naive | CLEC4A | Macrophages.M2 | PGF | Endothelial cells |
| ZNF204P | T.cells.CD4.naive | CRYBB1 | Macrophages.M2 | PLXNA2 | Endothelial cells |
| ZNF324 | T.cells.CD4.naive | FRMD4A | Macrophages.M2 | PTPRB | Endothelial cells |
| EPB41 | T.cells.CD4.memory.resting | HRH1 | Macrophages.M2 | ROBO4 | Endothelial cells |
| ETS1 | T.cells.CD4.memory.resting | MS4A6A | Macrophages.M2 | SDPR | Endothelial cells |
| FBXL8 | T.cells.CD4.memory.resting | NME8 | Macrophages.M2 | SHANK3 | Endothelial cells |
| RCAN3 | T.cells.CD4.memory.resting | NPL | Macrophages.M2 | SHE | Endothelial cells |
| RPL10L | T.cells.CD4.memory.resting | RENBP | Macrophages.M2 | TEK | Endothelial cells |
| TRAV13-2 | T.cells.CD4.memory.resting | WNT5B | Macrophages.M2 | TIE1 | Endothelial cells |
| CDC25A | T.cells.CD4.memory.activated | ALOX15 | Dendritic.cells.resting | VEPH1 | Endothelial cells |
| IFNG | T.cells.CD4.memory.activated | C1orf54 | Dendritic.cells.resting | VWF | Endothelial cells |
| IL17A | T.cells.CD4.memory.activated | CD1A | Dendritic.cells.resting | COL1A1 | Fibroblasts |
| IL26 | T.cells.CD4.memory.activated | CD1B | Dendritic.cells.resting | COL3A1 | Fibroblasts |
| IL3 | T.cells.CD4.memory.activated | CD1C | Dendritic.cells.resting | COL6A1 | Fibroblasts |
| IL4 | T.cells.CD4.memory.activated | CD1E | Dendritic.cells.resting | COL6A2 | Fibroblasts |
| IL9 | T.cells.CD4.memory.activated | DHRS11 | Dendritic.cells.resting | DCN | Fibroblasts |
| ORC1 | T.cells.CD4.memory.activated | MMP12 | Dendritic.cells.resting | GREM1 | Fibroblasts |
| RRP9 | T.cells.CD4.memory.activated | PPFIBP1 | Dendritic.cells.resting | PAMR1 | Fibroblasts |
| SKA1 | T.cells.CD4.memory.activated | RNASE6 | Dendritic.cells.resting | TAGLN | Fibroblasts |
| CHI3L2 | T.cells.follicular.helper | SCN9A | Dendritic.cells.resting | CCR2 | MDSC |
| CXCL13 | T.cells.follicular.helper | TREM2 | Dendritic.cells.resting | CD14 | MDSC |
| CXCR5 | T.cells.follicular.helper | ARHGAP22 | Dendritic.cells.activated | CD2 | MDSC |
| FZD3 | T.cells.follicular.helper | BIRC3 | Dendritic.cells.activated | CD86 | MDSC |
| ICA1 | T.cells.follicular.helper | CCL17 | Dendritic.cells.activated | CXCR4 | MDSC |
| ICOS | T.cells.follicular.helper | CCL22 | Dendritic.cells.activated | FCGR2A | MDSC |
| IL21 | T.cells.follicular.helper | CD86 | Dendritic.cells.activated | FCGR2B | MDSC |
| MAP4K1 | T.cells.follicular.helper | CHST7 | Dendritic.cells.activated | FCGR3A | MDSC |
| PASK | T.cells.follicular.helper | CLIC2 | Dendritic.cells.activated | FERMT3 | MDSC |
| PDCD1 | T.cells.follicular.helper | ETV3 | Dendritic.cells.activated | GPSM3 | MDSC |
| SLC7A10 | T.cells.follicular.helper | HTR2B | Dendritic.cells.activated | IL18BP | MDSC |
| ST8SIA1 | T.cells.follicular.helper | IL12B | Dendritic.cells.activated | IL4R | MDSC |
| TRIB2 | T.cells.follicular.helper | MAP3K13 | Dendritic.cells.activated | ITGAL | MDSC |
| TSHR | T.cells.follicular.helper | PDCD1LG2 | Dendritic.cells.activated | ITGAM | MDSC |
| ZAP70 | T.cells.follicular.helper | ADAMTS3 | Mast.cells.resting | PARVG | MDSC |
| ZBTB10 | T.cells.follicular.helper | ADRB2 | Mast.cells.resting | PSAP | MDSC |
| BARX2 | T.cells.regulatory..Tregs. | FAM124B | Mast.cells.resting | PTGER2 | MDSC |
| CD5 | T.cells.regulatory..Tregs. | FAM174B | Mast.cells.resting | PTGES2 | MDSC |
| CD7 | T.cells.regulatory..Tregs. | GFI1 | Mast.cells.resting | S100A8 | MDSC |
| CD70 | T.cells.regulatory..Tregs. | HOXA1 | Mast.cells.resting | S100A9 | MDSC |
| CEMP1 | T.cells.regulatory..Tregs. | MS4A2 | Mast.cells.resting | AIF1 | Macrophage |
| CRISP3 | T.cells.regulatory..Tregs. | GADD45B | Mast.cells.resting | CCL1 | Macrophage |
| CTLA4 | T.cells.regulatory..Tregs. | AZU1 | Mast.cells.activated | CCL14 | Macrophage |
| EFNA5 | T.cells.regulatory..Tregs. | CCL1 | Mast.cells.activated | CCL23 | Macrophage |
| FOXP3 | T.cells.regulatory..Tregs. | CCL20 | Mast.cells.activated | CCL26 | Macrophage |
| FRMD8 | T.cells.regulatory..Tregs. | CXCL3 | Mast.cells.activated | CD300LB | Macrophage |
| HIC1 | T.cells.regulatory..Tregs. | IL1B | Mast.cells.activated | CNR1 | Macrophage |
| HMGB3P30 | T.cells.regulatory..Tregs. | IL5 | Mast.cells.activated | CNR2 | Macrophage |
| KIRREL | T.cells.regulatory..Tregs. | LINC00597 | Mast.cells.activated | EIF1 | Macrophage |
| LAIR2 | T.cells.regulatory..Tregs. | ARVCF | Mast.cells.activated | EIF4A1 | Macrophage |
| LILRA4 | T.cells.regulatory..Tregs. | NOX3 | Mast.cells.activated | FPR1 | Macrophage |
| LOC126987 | T.cells.regulatory..Tregs. | NTRK1 | Mast.cells.activated | FPR2 | Macrophage |
| NPAS1 | T.cells.regulatory..Tregs. | TEC | Mast.cells.activated | FRAT2 | Macrophage |
| NTN3 | T.cells.regulatory..Tregs. | BCL2A1 | Eosinophils | GPR27 | Macrophage |
| PLCH2 | T.cells.regulatory..Tregs. | C3AR1 | Eosinophils | GPR77 | Macrophage |
| PMCH | T.cells.regulatory..Tregs. | CCR3 | Eosinophils | RNASE2 | Macrophage |
| RYR1 | T.cells.regulatory..Tregs. | CLC | Eosinophils | MS4A2 | Macrophage |
| SEC31B | T.cells.regulatory..Tregs. | DACH1 | Eosinophils | BASP1 | Macrophage |
| KLHL22 | T.cells.regulatory..Tregs. | DAPK2 | Eosinophils | IGSF6 | Macrophage |
| SIT1 | T.cells.regulatory..Tregs. | DEPDC5 | Eosinophils | HK3 | Macrophage |
| SKAP1 | T.cells.regulatory..Tregs. | EMR1 | Eosinophils | VNN1 | Macrophage |
| SSX1 | T.cells.regulatory..Tregs. | EMR3 | Eosinophils | FES | Macrophage |
| TRAV21 | T.cells.regulatory..Tregs. | EPN2 | Eosinophils | NPL | Macrophage |
| TRAV8-6 | T.cells.regulatory..Tregs. | GIPR | Eosinophils | FZD2 | Macrophage |
| TRAV9-2 | T.cells.regulatory..Tregs. | GPR183 | Eosinophils | FAM198B | Macrophage |
| TYR | T.cells.regulatory..Tregs. | GPR65 | Eosinophils | HNMT | Macrophage |
| CDH12 | T.cells.gamma.delta | GPR97 | Eosinophils | SLC15A3 | Macrophage |
| GZMK | T.cells.gamma.delta | IL5RA | Eosinophils | CD4 | Macrophage |
| TARDBPP1 | T.cells.gamma.delta | LRMP | Eosinophils | TXNDC3 | Macrophage |
| TRDC | T.cells.gamma.delta | NR4A3 | Eosinophils | FRMD4A | Macrophage |
| ZNF442 | T.cells.gamma.delta | OSM | Eosinophils | CRYBB1 | Macrophage |
| CDHR1 | NK.cells.resting | P2RY10 | Eosinophils | HRH1 | Macrophage |
| DEFA4 | NK.cells.resting | P2RY14 | Eosinophils | WNT5B | Macrophage |

**Supplementary Table S3. Markers of M2-TAM signature**

| ADK | CCL18 | CD209 | CTSS | GAS7 | IL10 | MS4A6A | SLC38A6 |
| --- | --- | --- | --- | --- | --- | --- | --- |
| ALOX15 | CCL22 | CD302 | CXCR4 | HEXB | IL1R2 | MSR1 | SLC4A7 |
| ARG1 | CCL23 | CERK | EGR2 | HNMT | IL1RN | P2RY13 | SLCO2B1 |
| CA2 | CCL24 | CHN2 | F13A1 | HRH1 | LIPA | P2RY14 | STAB1 |
| CCL13 | CCR2 | CLEC4F | FCER2 | HS3ST1 | LTA4H | P2RY5 | TGFB1 |
| CCL16 | CD14 | CLEC7A | FGL2 | HS3ST2 | MAF | RETNLB | TGFBR2 |
| CCL17 | CD163 | CTSC | FN1 | IGF1 | MMP9 | SEPP1 | TLR5 |
|  |  |  |  |  |  |  | TPST2 |
