# Supplementary figures and images for: Tumor cell-derived SPON2 promotes M2-polarized tumor-associated macrophage infiltration and cancer progression by activating PYK2 in CRC
Source: J Exp Clin Cancer Res. 2021 Sep 28;40:304. doi: 10.1186/s13046-021-02108-0 (PMC8477524; doi:10.1186/s13046-021-02108-0)

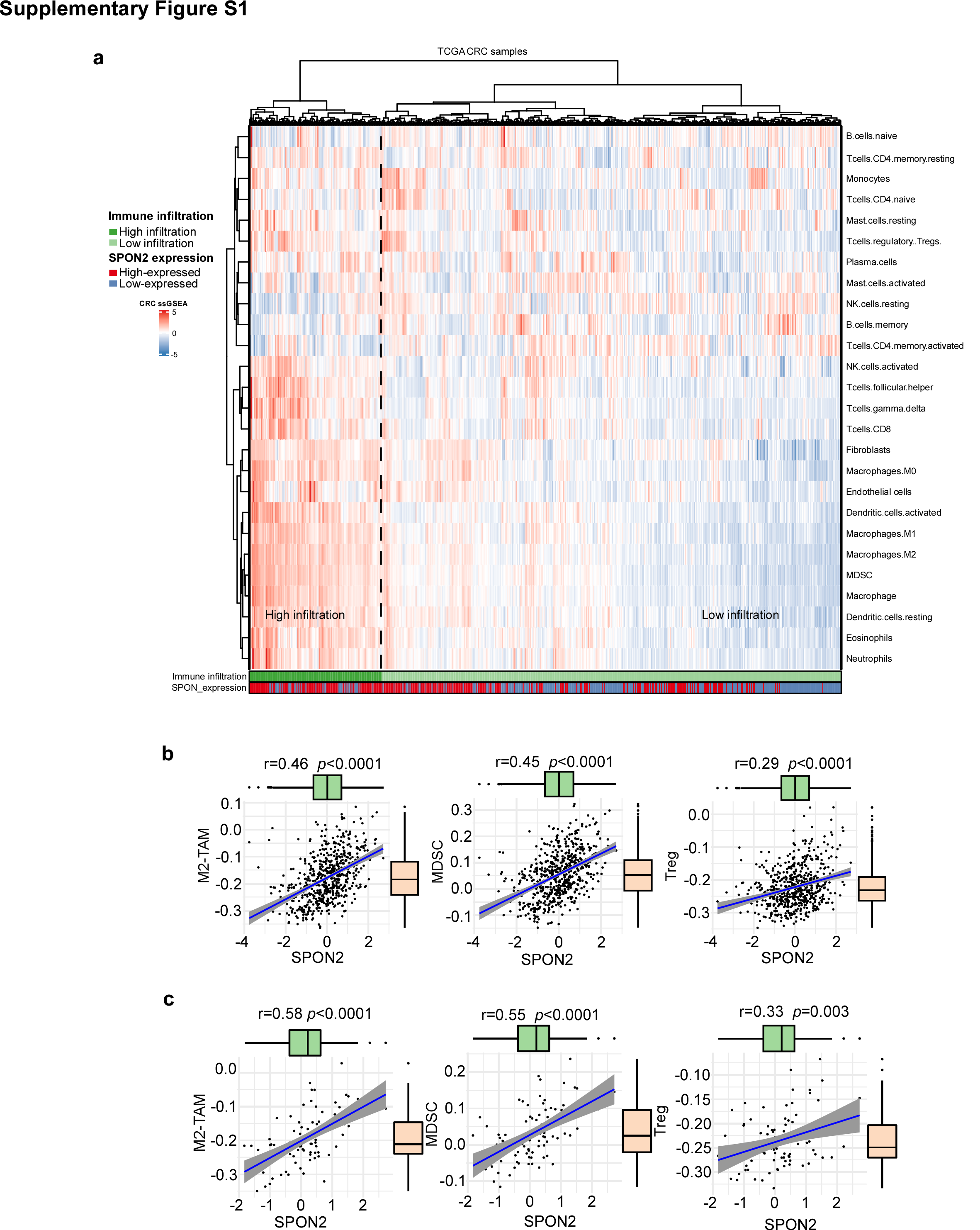

Supplement: Supplementary file 1 — Additional file 1: Supplementary Figure S1. Correlation between SPON2 expression and immune cell signatures in TCGA COAD and READ data. a. The heat map of the expression of immune cells between high- and low-expressed SPON2. b. The Pearson correlation between the expression of immune cells and SPON2 mRNA expression level in all sample of TCGA COAD and READ. c. The Pearson correlation between the expression of immune cells and SPON2 mRNA expression level in all sample with Stage IV. Supplementary Figure S2. SPON2 derived from CRC cells promotes TAMs migration and infiltration of MDSCs and Tregs in tumors. a. Western blot of SPON2 protein levels in the cell lysates and conditioned media of colorectal cancer cell lines. b. Western blot for the expression of SPON2 protein in whole cell lysate (WC) and conditioned medium (CM) of SW480/Vector, SW480/SPON2, SW620/Scramble, SW620/shSPON2#1 and SW620/shSPON2#2. c. Western blot for the expression of SPON2 protein in whole cell lysate (WC) and conditioned medium (CM) of MC38/Vector, MC38/SPON2, MC38/Scramble, MC38/shSpon2#1.and MC38/shSpon2#2. d. Migration of RAW264.7 cells toward conditioned medium from stable cell lines. Scale bar, 100 μm. e. FACS plot showing percentage of MDSCs (CD45+/CD11b+/F4/80-/Gr-1+) in orthotopic tumors from MC38/Scramble and MC38/shSpon2#1. f. FACS plot showing percentage of Tregs (CD45+/CD3e+/CD4+/FoxP3+) in orthotopic tumors from MC38/Scramble and MC38/shSpon2#1. Supplementary Figure S3. Flow cytometry analysis of the proportion of M2-like cells in M0 macrophages, M2 macrophages, and M0 macrophages co-cultured with MC38/Scramble, MC38/shSpon2#1and MC38/shSpon2#2 cell lines. Supplementary Figure S4. Tumor weights and infiltration of TAMs. a. Tumor weights of mice in the different treatment groups. b. Flow cytometry gating strategy for TAMs (CD45+, CD11b+, F4/80+) showing the efficiency of macrophage depletion. Supplementary Figure S5. SPON2 promotes monocyte transendothelial migration and tumor gro [file 13046_2021_2108_MOESM1_ESM.zip › new-sFigure 1.tif]

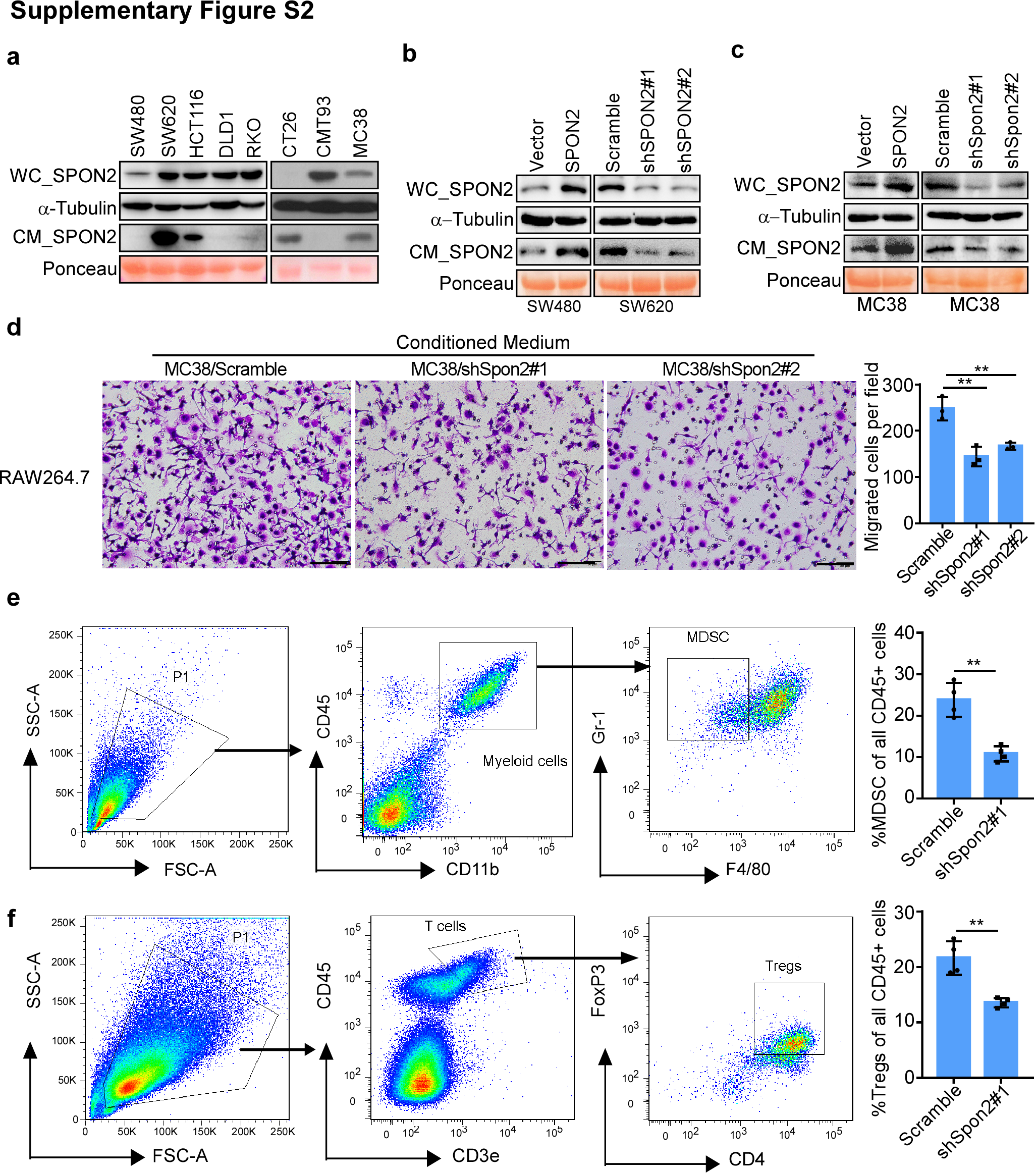

Supplement: Supplementary file 1 — Additional file 1: Supplementary Figure S1. Correlation between SPON2 expression and immune cell signatures in TCGA COAD and READ data. a. The heat map of the expression of immune cells between high- and low-expressed SPON2. b. The Pearson correlation between the expression of immune cells and SPON2 mRNA expression level in all sample of TCGA COAD and READ. c. The Pearson correlation between the expression of immune cells and SPON2 mRNA expression level in all sample with Stage IV. Supplementary Figure S2. SPON2 derived from CRC cells promotes TAMs migration and infiltration of MDSCs and Tregs in tumors. a. Western blot of SPON2 protein levels in the cell lysates and conditioned media of colorectal cancer cell lines. b. Western blot for the expression of SPON2 protein in whole cell lysate (WC) and conditioned medium (CM) of SW480/Vector, SW480/SPON2, SW620/Scramble, SW620/shSPON2#1 and SW620/shSPON2#2. c. Western blot for the expression of SPON2 protein in whole cell lysate (WC) and conditioned medium (CM) of MC38/Vector, MC38/SPON2, MC38/Scramble, MC38/shSpon2#1.and MC38/shSpon2#2. d. Migration of RAW264.7 cells toward conditioned medium from stable cell lines. Scale bar, 100 μm. e. FACS plot showing percentage of MDSCs (CD45+/CD11b+/F4/80-/Gr-1+) in orthotopic tumors from MC38/Scramble and MC38/shSpon2#1. f. FACS plot showing percentage of Tregs (CD45+/CD3e+/CD4+/FoxP3+) in orthotopic tumors from MC38/Scramble and MC38/shSpon2#1. Supplementary Figure S3. Flow cytometry analysis of the proportion of M2-like cells in M0 macrophages, M2 macrophages, and M0 macrophages co-cultured with MC38/Scramble, MC38/shSpon2#1and MC38/shSpon2#2 cell lines. Supplementary Figure S4. Tumor weights and infiltration of TAMs. a. Tumor weights of mice in the different treatment groups. b. Flow cytometry gating strategy for TAMs (CD45+, CD11b+, F4/80+) showing the efficiency of macrophage depletion. Supplementary Figure S5. SPON2 promotes monocyte transendothelial migration and tumor gro [file 13046_2021_2108_MOESM1_ESM.zip › new-sFigure 2.tif]

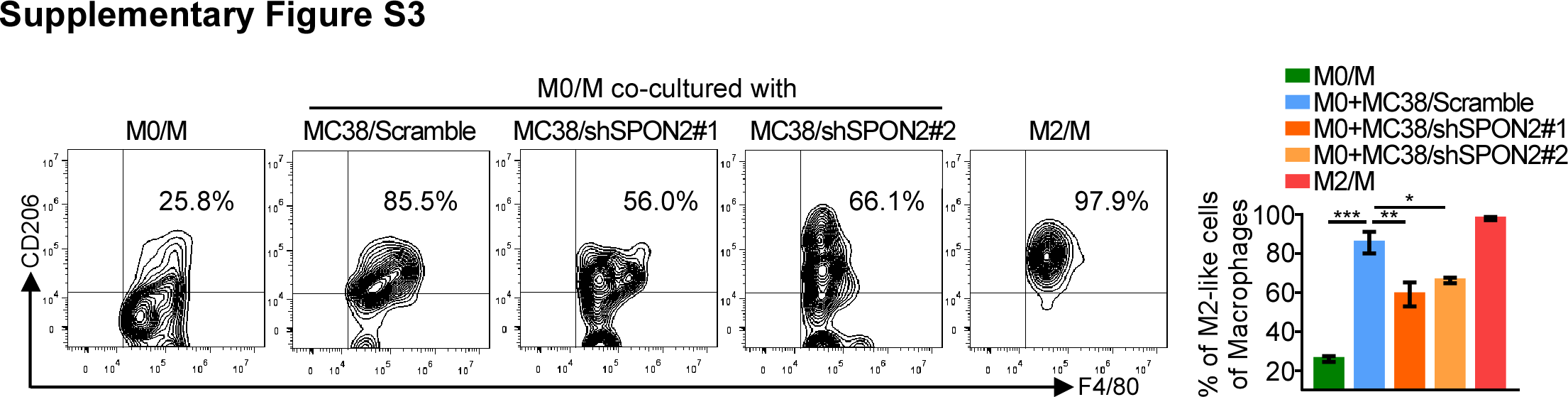

Supplement: Supplementary file 1 — Additional file 1: Supplementary Figure S1. Correlation between SPON2 expression and immune cell signatures in TCGA COAD and READ data. a. The heat map of the expression of immune cells between high- and low-expressed SPON2. b. The Pearson correlation between the expression of immune cells and SPON2 mRNA expression level in all sample of TCGA COAD and READ. c. The Pearson correlation between the expression of immune cells and SPON2 mRNA expression level in all sample with Stage IV. Supplementary Figure S2. SPON2 derived from CRC cells promotes TAMs migration and infiltration of MDSCs and Tregs in tumors. a. Western blot of SPON2 protein levels in the cell lysates and conditioned media of colorectal cancer cell lines. b. Western blot for the expression of SPON2 protein in whole cell lysate (WC) and conditioned medium (CM) of SW480/Vector, SW480/SPON2, SW620/Scramble, SW620/shSPON2#1 and SW620/shSPON2#2. c. Western blot for the expression of SPON2 protein in whole cell lysate (WC) and conditioned medium (CM) of MC38/Vector, MC38/SPON2, MC38/Scramble, MC38/shSpon2#1.and MC38/shSpon2#2. d. Migration of RAW264.7 cells toward conditioned medium from stable cell lines. Scale bar, 100 μm. e. FACS plot showing percentage of MDSCs (CD45+/CD11b+/F4/80-/Gr-1+) in orthotopic tumors from MC38/Scramble and MC38/shSpon2#1. f. FACS plot showing percentage of Tregs (CD45+/CD3e+/CD4+/FoxP3+) in orthotopic tumors from MC38/Scramble and MC38/shSpon2#1. Supplementary Figure S3. Flow cytometry analysis of the proportion of M2-like cells in M0 macrophages, M2 macrophages, and M0 macrophages co-cultured with MC38/Scramble, MC38/shSpon2#1and MC38/shSpon2#2 cell lines. Supplementary Figure S4. Tumor weights and infiltration of TAMs. a. Tumor weights of mice in the different treatment groups. b. Flow cytometry gating strategy for TAMs (CD45+, CD11b+, F4/80+) showing the efficiency of macrophage depletion. Supplementary Figure S5. SPON2 promotes monocyte transendothelial migration and tumor gro [file 13046_2021_2108_MOESM1_ESM.zip › new-sFigure 3.tif]

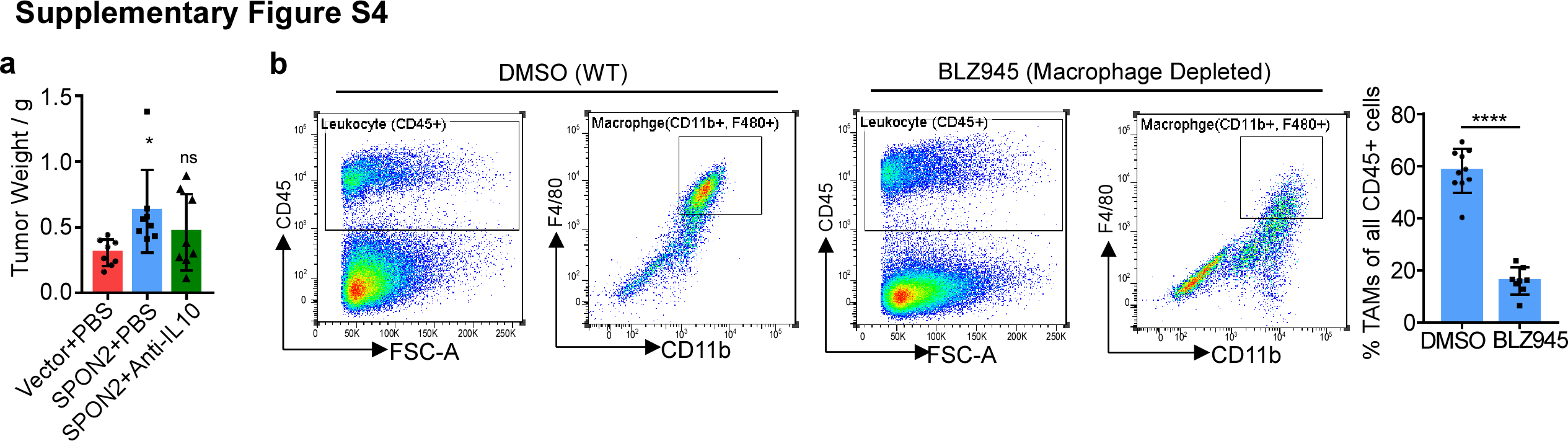

Supplement: Supplementary file 1 — Additional file 1: Supplementary Figure S1. Correlation between SPON2 expression and immune cell signatures in TCGA COAD and READ data. a. The heat map of the expression of immune cells between high- and low-expressed SPON2. b. The Pearson correlation between the expression of immune cells and SPON2 mRNA expression level in all sample of TCGA COAD and READ. c. The Pearson correlation between the expression of immune cells and SPON2 mRNA expression level in all sample with Stage IV. Supplementary Figure S2. SPON2 derived from CRC cells promotes TAMs migration and infiltration of MDSCs and Tregs in tumors. a. Western blot of SPON2 protein levels in the cell lysates and conditioned media of colorectal cancer cell lines. b. Western blot for the expression of SPON2 protein in whole cell lysate (WC) and conditioned medium (CM) of SW480/Vector, SW480/SPON2, SW620/Scramble, SW620/shSPON2#1 and SW620/shSPON2#2. c. Western blot for the expression of SPON2 protein in whole cell lysate (WC) and conditioned medium (CM) of MC38/Vector, MC38/SPON2, MC38/Scramble, MC38/shSpon2#1.and MC38/shSpon2#2. d. Migration of RAW264.7 cells toward conditioned medium from stable cell lines. Scale bar, 100 μm. e. FACS plot showing percentage of MDSCs (CD45+/CD11b+/F4/80-/Gr-1+) in orthotopic tumors from MC38/Scramble and MC38/shSpon2#1. f. FACS plot showing percentage of Tregs (CD45+/CD3e+/CD4+/FoxP3+) in orthotopic tumors from MC38/Scramble and MC38/shSpon2#1. Supplementary Figure S3. Flow cytometry analysis of the proportion of M2-like cells in M0 macrophages, M2 macrophages, and M0 macrophages co-cultured with MC38/Scramble, MC38/shSpon2#1and MC38/shSpon2#2 cell lines. Supplementary Figure S4. Tumor weights and infiltration of TAMs. a. Tumor weights of mice in the different treatment groups. b. Flow cytometry gating strategy for TAMs (CD45+, CD11b+, F4/80+) showing the efficiency of macrophage depletion. Supplementary Figure S5. SPON2 promotes monocyte transendothelial migration and tumor gro [file 13046_2021_2108_MOESM1_ESM.zip › new-sFigure 4.tif]

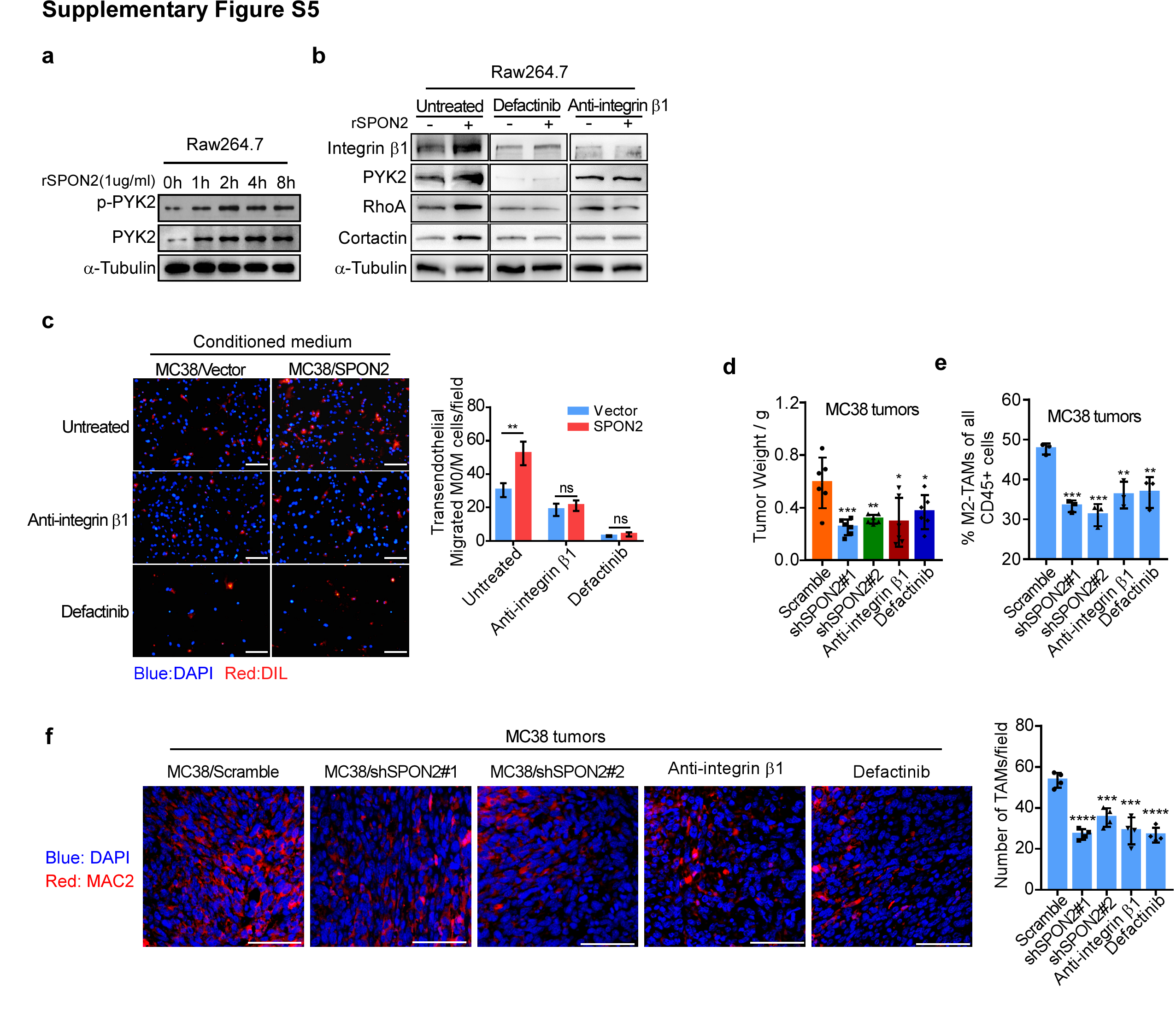

Supplement: Supplementary file 1 — Additional file 1: Supplementary Figure S1. Correlation between SPON2 expression and immune cell signatures in TCGA COAD and READ data. a. The heat map of the expression of immune cells between high- and low-expressed SPON2. b. The Pearson correlation between the expression of immune cells and SPON2 mRNA expression level in all sample of TCGA COAD and READ. c. The Pearson correlation between the expression of immune cells and SPON2 mRNA expression level in all sample with Stage IV. Supplementary Figure S2. SPON2 derived from CRC cells promotes TAMs migration and infiltration of MDSCs and Tregs in tumors. a. Western blot of SPON2 protein levels in the cell lysates and conditioned media of colorectal cancer cell lines. b. Western blot for the expression of SPON2 protein in whole cell lysate (WC) and conditioned medium (CM) of SW480/Vector, SW480/SPON2, SW620/Scramble, SW620/shSPON2#1 and SW620/shSPON2#2. c. Western blot for the expression of SPON2 protein in whole cell lysate (WC) and conditioned medium (CM) of MC38/Vector, MC38/SPON2, MC38/Scramble, MC38/shSpon2#1.and MC38/shSpon2#2. d. Migration of RAW264.7 cells toward conditioned medium from stable cell lines. Scale bar, 100 μm. e. FACS plot showing percentage of MDSCs (CD45+/CD11b+/F4/80-/Gr-1+) in orthotopic tumors from MC38/Scramble and MC38/shSpon2#1. f. FACS plot showing percentage of Tregs (CD45+/CD3e+/CD4+/FoxP3+) in orthotopic tumors from MC38/Scramble and MC38/shSpon2#1. Supplementary Figure S3. Flow cytometry analysis of the proportion of M2-like cells in M0 macrophages, M2 macrophages, and M0 macrophages co-cultured with MC38/Scramble, MC38/shSpon2#1and MC38/shSpon2#2 cell lines. Supplementary Figure S4. Tumor weights and infiltration of TAMs. a. Tumor weights of mice in the different treatment groups. b. Flow cytometry gating strategy for TAMs (CD45+, CD11b+, F4/80+) showing the efficiency of macrophage depletion. Supplementary Figure S5. SPON2 promotes monocyte transendothelial migration and tumor gro [file 13046_2021_2108_MOESM1_ESM.zip › new-sFigure 5.tif]
